# The mixed effects of online diversity training

**DOI:** 10.1073/pnas.1816076116

**Published:** 2019-04-01

**Authors:** Edward H. Chang, Katherine L. Milkman, Dena M. Gromet, Robert W. Rebele, Cade Massey, Angela L. Duckworth, Adam M. Grant

**Affiliations:** ^a^Department of Operations, Information, & Decisions, The Wharton School, University of Pennsylvania, Philadelphia, PA 19104;; ^b^Behavior Change for Good Initiative, University of Pennsylvania, Philadelphia, PA 19104;; ^c^Wharton People Analytics, The Wharton School, University of Pennsylvania, Philadelphia, PA 19104;; ^d^Melbourne School of Psychological Sciences, The University of Melbourne, Parkville, VIC, 3010 Australia;; ^e^Department of Psychology, University of Pennsylvania, Philadelphia, PA 19104;; ^f^Department of Management, The Wharton School, University of Pennsylvania, Philadelphia, PA 19104

**Keywords:** diversity training, gender, race, bias, field experiment

## Abstract

We present results from a large (*n* = 3,016) field experiment at a global organization testing whether a brief science-based online diversity training can change attitudes and behaviors toward women in the workplace. Our preregistered field experiment included an active placebo control and measured participants’ attitudes and real workplace decisions up to 20 weeks postintervention. Among groups whose average untreated attitudes—whereas still supportive of women—were relatively less supportive of women than other groups, our diversity training successfully produced attitude change but not behavior change. On the other hand, our diversity training successfully generated some behavior change among groups whose average untreated attitudes were already strongly supportive of women before training. This paper extends our knowledge about the pathways to attitude and behavior change in the context of bias reduction. However, the results suggest that the one-off diversity trainings that are commonplace in organizations are unlikely to be stand-alone solutions for promoting equality in the workplace, particularly given their limited efficacy among those groups whose behaviors policymakers are most eager to influence.

## Significance

Although diversity training is commonplace in organizations, the relative scarcity of field experiments testing its effectiveness leaves ambiguity about whether diversity training improves attitudes and behaviors toward women and racial minorities. We present the results of a large field experiment with an international organization testing whether a short online diversity training can affect attitudes and workplace behaviors. Although we find evidence of attitude change and some limited behavior change as a result of our training, our results suggest that the one-off diversity trainings that are commonplace in organizations are not panaceas for remedying bias in the workplace.

In 2018, two black men were arrested in a Philadelphia Starbucks after they asked to use the bathroom but declined to place an order while waiting for a friend (1). Starbucks’ response to this incident was to announce the closure of all US stores for an afternoon so employees could complete bias training (1). This fueled a national conversation about how organizations can prevent their employees from exhibiting bias and whether diversity training is an effective solution (2). Although more than half of midsized and large employers in the United States offer diversity training to their employees (3), it remains unclear whether diversity training improves attitudes and behaviors toward women and racial minorities given the lack of field experiments testing its effectiveness (4). In fact, one widely cited correlational study suggests diversity training may produce primarily negative outcomes for women and racial minorities in the workplace (5). We present the results of a large field experiment with an international organization testing whether a short (1-h) online diversity training can affect attitudes and workplace behaviors.

Recent meta-analyses suggest that diversity training can be effective with stronger effects on cognitive learning and weaker effects on attitudinal and behavioral measures, albeit with significant heterogeneity (6–8). These meta-analyses have also highlighted a number of factors that make diversity training more or less effective, such as whether there are other diversity-related initiatives in the organizational context (7). However, past studies have been subject to a number of limitations including difficulties in separating correlation from causation (5), a lack of behavioral outcomes for field experiments (9–11), and the inability to rule out demand effects or social desirability concerns (9, 11, 12). To overcome these limitations and advance knowledge about the value of diversity training, we conducted a preregistered field experiment with 3,016 participants at a large global organization, which included an active placebo control group and measured the effect of training on both attitudes and workplace behaviors. A particularly important innovation was the measurement of objective behavioral outcome variables that were not ostensibly connected to the training, addressing key concerns about whether demand effects bias the results of past diversity training research (7).

The training we tested drew on best practices and strategies for changing attitudes and behavior from interventions conducted in a wide range of other contexts. These strategies include targeting the specific underlying psychological process believed to produce undesirable outcomes (13), offering personalized feedback about individuals’ own biases to motivate change (14), destigmatizing attempts to improve on undesirable behaviors (15, 16), and offering actionable strategies for improvement and the opportunity to practice these strategies (10). Specifically, we designed the diversity training to raise awareness about the pervasiveness of stereotypes, share scientific evidence of the impact of stereotyping on important workplace behaviors, destigmatize and expose participants to their own stereotyping, provide evidence-based strategies for overcoming stereotyping, and allow employees to practice deploying evidence-based strategies to combat bias by responding to different workplace scenarios. Following recommendations from correlational research on diversity programs (17), this training was also voluntary.

## Diversity Training Experiment

We partnered with a large global organization to design and test our diversity training. The organization offered our training to employees as part of a broad strategic effort around inclusion and inclusive leadership. Our primary goal was to promote inclusive attitudes and behaviors toward women, whereas a secondary focus was to promote the inclusion of other under-represented groups (e.g., racial minorities). Our partner emailed 10,983 of their salaried employees worldwide in early 2017 to invite them to complete a new inclusive leadership workplace training. Through a 6-wk recruitment effort, 3,016 employees consented to be included in the research and began the hour-long training. These 3,016 employees (61.5% male; 38.5% located in the United States; 63 countries represented) were randomly assigned to one of three experimental conditions: a gender-bias training, a general-bias training, or a control training. In the gender-bias and general-bias trainings, hereafter referred to collectively as our treatment condition, participants learned about the psychological processes that underlie stereotyping and research that shows how stereotyping can result in bias and inequity in the workplace, completed and received feedback on an Implicit Association Test (18) assessing their associations between gender and career-oriented words, and learned about strategies to overcome stereotyping in the workplace. The gender-bias and general-bias trainings differed minimally: The gender-bias condition discussed only gender bias and stereotyping, whereas the general-bias condition included information on additional social categories (e.g., race and sexual orientation) in addition to gender. For simplicity of presentation, we collapse the gender-bias condition and general-bias condition in our primary analyses, but additional analyses separating out each condition (which differed minimally in effectiveness) are available in the *SI Appendix*. If anything, collapsing our treatment conditions weakens our primary results because the gender-bias condition was directionally more effective than the general-bias condition on most measures (*SI Appendix*, Tables S1–S3). Participants in our control condition received a stylistically similar training, but the focus was on psychological safety and active listening rather than stereotyping.

The median completion time for our online training was 68 min, and there was no differential attrition between the treatment and the control conditions (*z* = 0.47; *P* = 0.64). We measured the effectiveness of our diversity training in several ways, following guidelines from past diversity research (19). First, we measured attitudes at the end of our training via survey questions. Second, we unobtrusively measured real workplace behaviors with no ostensible connection to our intervention for several months following the training.

In our preanalysis plan (*SI Appendix*), we specified that we would use controlled ordinary least-squares regressions with interaction terms to estimate overall treatment effects and analyze treatment effect differences between men and women and between employees located inside and outside the United States. In exploratory analyses, we examined differences in treatment effects for men and women based on their country location, and we added interaction terms to estimate treatment effect differences between whites and racial minorities in the United States when examining outcomes pertaining to racial minorities. All statistics reported here use Wald tests to calculate treatment effects from these regressions (see *SI Appendix* for details on our regression models as well as *t* tests comparing conditions, which yield essentially the same results). When analyzing attitudinal measures, we standardize them for ease of interpretation. Whenever possible, we analyze data on an intent-to-treat basis, which means we analyze data from all participants randomized into a condition, regardless of whether they completed the entire training (20). We find the same results when we limit analyses only to employees who completed the entire training (*SI Appendix*).

## Results

### Effects of Diversity Training on Attitudes and Behaviors Toward Women.

**Fig. 1.**
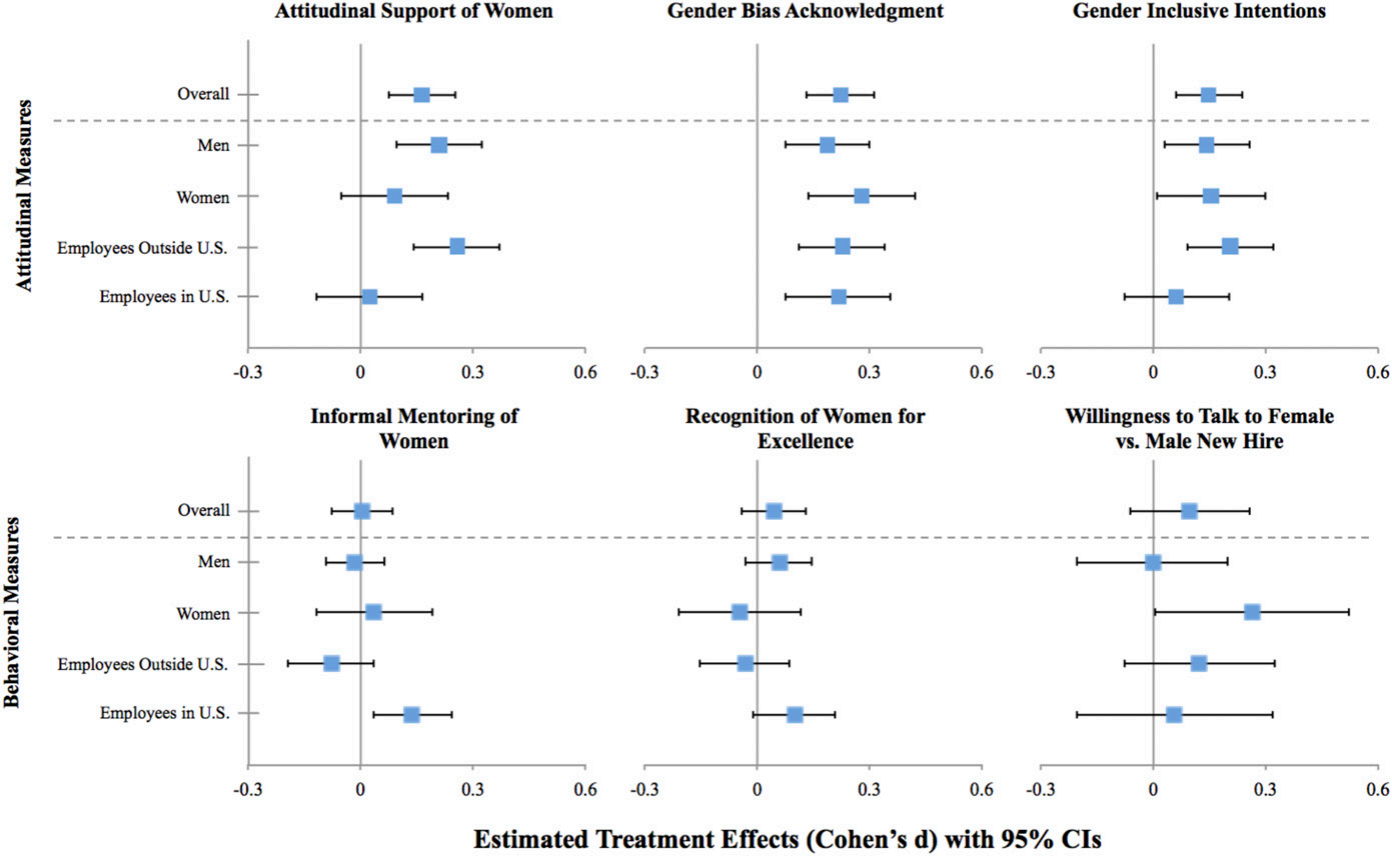
Summary of the intervention’s effect on outcome measures. Note: This figure summarizes the intervention’s effect on each of the attitude and behavior measures collected. Treatment effects (Cohen’s d) are estimated from ordinary least-squares regressions predicting the specified outcome measure using all interactions between the treatment, an indicator for the participant being male, and an indicator for the participant being located in the United States and fixed effects for office location, job category, and race. Mean differences are estimated via Wald tests, whereas pooled SDs are estimated via the root mean squared error from the regressions. Error bars reflect 95% confidence intervals.

Our diversity training had a significant positive effect on employees’ attitudes toward women across all measures collected. First, we adapted a validated scale (21) to assess employees’ attitudinal support for women (i.e., willingness to acknowledge discrimination against women and support for policies designed to help women). We found that the treatment had a significant positive effect on employees’ attitudinal support for women (*b* = 0.149, *P* < 0.001). We also conducted preregistered subgroup analyses to determine whether this effect was driven by particular subgroups of participants. As seen in Fig. 1, this effect was driven by international employees: employees outside the United States who completed the diversity training reported greater attitudinal support for women than those who completed our control training (*b* = 0.234, *P* < 0.001), whereas employees in the United States did not (*b* = 0.0212, *P* = 0.747), and this difference was significant (*P* = 0.012).

Our second attitudinal measure examined differences in employees’ perceptions of other people’s gender biases compared with their own, following previous work on the bias blind spot that suggests that people believe they are less biased than others (22). We found that our treatment had a significant positive effect on employees’ willingness to acknowledge that their own gender biases matched those of the general population (*b* = 0.217, *P* < 0.001), and all preregistered subgroups showed significant treatment effects (smallest *b* = 0.181, all *P*’s < 0.05; see Fig. 1).

Our third and final attitudinal measure was a situational judgment test created and validated to measure intentions to engage in inclusive workplace behaviors toward women (*SI Appendix*). To assess behavioral intentions in situations where bias or stereotyping may arise, employees were presented with different realistic workplace scenarios created in collaboration with our organizational partner. From a list of options, they then indicated how they were most and least likely to behave. Higher scores on this measure represent stronger behavioral intentions to be inclusive toward women in the workplace. We found that the treatment had a significant positive effect on gender-inclusive intentions (*b* = 0.147, *P* = 0.001). In our preregistered subgroup analyses, we found that this overall treatment effect was driven by employees outside the United States as they showed a significant treatment effect (*b* = 0.206, *P* = 0.001), whereas employees in the United States did not (*b* = 0.0614, *P* = 0.392; see Fig. 1).

Together, these results suggest diversity training can have a significant positive effect on attitudes toward women in the workplace. However, these effects were particularly concentrated among employees outside the United States, whose attitudes in the absence of intervention—whereas still supportive of women—were less supportive than those of US employees (attitudinal support for women in the control group: ∆_US_ _employees_minus_international_ _employees_ = 0.622, *t*(781) = 8.47, *P* < 0.0001; see *SI Appendix*) and therefore had more room to improve. Although employees in the United States had more supportive attitudes toward women than employees outside the United States, the average attitudinal support for women among employees outside the United States was still well above the midpoint of the scale.

To assess the impact of our training on workplace behaviors, we collected three measures. First, 3 wk after the recruitment period for inclusive leadership training ended, our organizational partner emailed employees about a new initiative designed to foster inclusiveness in the workplace. Employees were invited to nominate up to five colleagues to informally mentor over coffee through this initiative. This was a real workplace program that had no ostensible connection to our training. We measured the average number of women selected per employee for informal mentoring through this initiative by condition. In our preregistered subgroup analyses, we found that, although our treatment did not have a significant positive effect on the number of women selected as mentees overall (*b* = 0.00288, *P* = 0.914), it did have a significant positive effect on the number of women selected as mentees by US employees (*b* = 0.0902, *P* = 0.011) as seen in Fig. 1. More specifically, this was driven by female employees in the United States (*b* = 0.203, *P* = 0.001), who showed a significantly larger treatment effect than any other group (all *P*’s < 0.004). Interestingly, in exploratory analyses, we found that this result was driven by women in the United States using the program to seek out informal mentorship from senior colleagues regardless of gender (*P* < 0.001), although the treatment also had a marginal positive effect on their mentoring of junior female colleagues (*P* = 0.067; see *SI Appendix*). Thus, our intervention appears to have prompted women in the United States to both engage in more inclusive behaviors toward women and take more initiative in overcoming any potential obstacles or barriers they face in the workplace. These estimates suggest that for every five women in the United States assigned to the treatment condition (as opposed to the control condition), an additional woman was invited out to coffee for a mentoring meeting.

Three weeks later (see *SI Appendix*, Fig. S1 for a study timeline), our field partner emailed employees soliciting nominations to recognize a colleague’s excellence. Again, this was a real workplace program with no ostensible connection to our training. We measured the average number of women recognized per employee by condition. Our treatment did not have a significant effect on the number of women nominated overall (*b* = 0.00213, *P* = 0.687), but in our preregistered subgroup analyses, we found that it did produce a marginal positive effect on the number of women recognized for excellence by US employees (*b* = 0.0121, *P* = 0.075; see Fig. 1).

Finally, 14 wk after the recruitment period for the training had ended, our field partner emailed employees asking if they would be willing to spend 15 min on the phone with a male new hire or a female new hire (randomly assigned) in an audit study design (23). We measured the difference in sign-ups to talk with a female new hire relative to a male new hire by condition. Our treatment did not have a significant effect on the difference in the overall signup rate to speak with a female or male new hire (*b* = 0.0467, *P* = 0.234), but in our preregistered subgroup analyses, we found it did have a significant effect among female employees, leading them to favor speaking with a female new hire over a male new hire (*b* = 0.127; *P* = 0.047; see Fig. 1).

Overall, these results paint a less consistent picture of the benefits of diversity training as a means of influencing workplace behaviors toward women than as a method for improving attitudes. However, we do find some evidence of benefits. In particular, the behavior change we observed was concentrated among those groups (e.g., women in the United States) who had the most supportive attitudes toward women in the absence of intervention. This is an interesting contrast to our findings regarding attitude change: Attitude change was concentrated among relatively less supportive groups. Exploratory analyses breaking down our data into 86 country-gender subgroups offer additional support for the conclusion that our diversity training’s effects were moderated by a group’s attitudes in the absence of treatment (*SI Appendix*). Specifically, we found that attitudinal support for women increased more in response to the training among employees in subgroups whose pretraining attitudes were relatively less supportive of women (*P* < 0.001; see *SI Appendix*, Table S22). On the other hand, the training’s effect on our most sensitive behavioral outcome—inviting more women to connect over coffee—was larger for employees in subgroups whose pretraining attitudes were relatively more supportive of women (*P* = 0.012; see *SI Appendix*, Table S22).

### Effects of Diversity Training on Attitudes and Behaviors Toward Racial Minorities.

Although our primary aim was to assess the impact of diversity training on attitudes and behaviors toward women, we also collected some data on participants’ postintervention attitudes and behaviors toward racial minorities. We collected attitudinal data for all employees, but our organizational partner only tracks the race of US employees, so we could only measure behavioral outcomes pertaining to the inclusion of racial minorities in the United States. We found a significant main effect of diversity training on employees’ willingness to acknowledge the extent to which their own personal racial biases matched the racial biases of the general population (*b* = 0.193, *P* < 0.001), which was our sole attitudinal measure addressing racial bias. We also found a marginal positive overall effect of treatment on the number of racial minorities selected for informal mentoring (*b* = 0.0470, *P* = 0.052) and a significant positive effect of treatment on the number of racial minorities recognized for excellence (*b* = 0.0170, *P* = 0.039) in the United States. Paralleling our gender findings, in exploratory analyses, we found that the treatment effect on racial minorities recognized for excellence was driven by racial minority employees (treatment effect for whites: *b* = −0.00204, *P* = 0.738; treatment effect for racial minorities: *b* = 0.0449, *P* = 0.006), and this difference was significant (*P* = 0.004).

We also assessed the gender-bias training on its own to examine whether this gender-focused training (which did not reference racial bias or stereotyping) could spill over to benefit other under-represented groups. There were significant positive effects of the gender-bias training on the number of racial minorities selected for informal mentoring (*b* = 0.0539, *P* = 0.044), the number of racial minorities recognized for excellence (*b* = 0.0260, *P* = 0.016), and employees’ willingness to acknowledge that their own racial biases matched the racial biases of the general population (*b* = 0.169, *P* = 0.002). These results suggest there can be beneficial spillover effects from bias reduction efforts since a diversity training focused exclusively on gender bias and stereotyping positively affected employees’ attitudes and behaviors toward racial minorities in the workplace (see *SI Appendix* for results broken out for the general-bias condition). Interestingly, these spillovers appear to be driven by members of historically disadvantaged groups in the United States, consistent with past research and theorizing on stigma-based solidarity (24).

## Discussion

Our field experiment testing the efficacy of a diversity training intervention provides important new insights about who will respond to interventions and why, shedding light on how behavior change comes about. Past theory has conceptualized behavior as originating from attitudes, which develop into intentions, and finally shape actions (25). Our findings are consistent with this model of behavior change. Specifically, our diversity training generated more behavior change—and less attitude change—among subgroups whose average untreated attitudes were strongly supportive of women. On the other hand, our diversity training produced less behavior change and more attitude change in groups whose average untreated attitudes were relatively less supportive of women. For both results, we find this pattern in preregistered analyses comparing men and women inside and outside of the United States and in exploratory analyses of 86 country-gender subgroups.

This model of behavior change accords with empirical evidence from interventions conducted in other settings. For example, interventions designed to change students’ academic mindsets to be less fixed have found that the largest changes in mindsets occur among those with fixed mindsets preintervention (26), and interventions to increase water conservation show the largest behavioral effects among participants with the most environmentally friendly attitudes preintervention (27). Our paper suggests the effectiveness of diversity training may depend on the audience and their preexisting attitudes.

That said, the robust effects of our hour-long online diversity training on self-reported attitudes, coupled with the detection of some changes in real workplace behaviors in the weeks post-training, offer some encouraging news for advocates of diversity training. They suggest that even brief online diversity training interventions can create some value. We also find no evidence of backlash or reactance against our diversity training, perhaps in part because training was voluntary and the population studied had relatively progressive baseline gender attitudes.

However, there are many reasons to be skeptical about the value of brief diversity trainings in light of our findings. First, because we measured attitudes via surveys at the end of training, it is possible that our results on attitude change are driven in part by demand effects or social desirability. We cannot rule out these alternative explanations for the attitude shifts measured, so future research should measure attitudes over longer time periods to mitigate these concerns. Second, we see mostly null effects when it comes to behavior change, and the subpopulations who did change some of their behaviors (i.e., women in the United States, racial minorities) were not the subgroups policymakers typically hope to influence most with such interventions. Although it is encouraging that we observe any lasting behavior change in response to a short online training program, the lack of change in the behaviors among dominant group members indicates that additional remedies are needed to improve the overall work experiences of women and racial minorities.

Notably, we conducted our training in only one organization, which is a limitation, as the effects of diversity training are likely to be context specific. In addition, we focused primarily on measuring inclusive behaviors, compared with other studies which have focused on other kinds of outcomes, such as changes in the representation of women and minorities in management positions (5). Based on our proposed model of behavior change, we might expect diversity training to have stronger effects on attitudes but weaker effects on behaviors in other organizations where employees have relatively less supportive attitudes toward women. Additional field experiments testing the effectiveness of diversity training in other settings would be valuable.

Even if brief diversity training interventions do not influence behavior for many groups, other benefits of diversity training may exist that we did not measure. For instance, offering diversity training may signal that diversity is valued, and trainings may also set norms of inclusion. Both the establishment of social norms and the signaling of organizational values may lead to benefits for women and racial minorities in the workplace, and future research should measure such potential benefits.

Interestingly, one of the strongest behavioral effects we detect is that our training prompted women to connect with more senior women. Given that our training highlighted research documenting bias and stereotyping against women in the workplace, our training may have signaled to women that they needed to be more proactive about their advancement in the company. Further research attempting to replicate the finding that diversity training motivates under-represented groups to be more proactive would be valuable.

Overall, our research suggests that more effortful interventions may be needed to robustly change employee behavior. For example, organizations could devote resources to recruit more women and under-represented minorities (28), particularly into leadership roles (29), or change processes and structures to mitigate the effects of stereotyping and bias (30). Trainings that are much more involved than the one we tested (e.g., programs that employees engage with repeatedly over many weeks or months) should also be empirically evaluated. It may be too much to expect short one-off diversity trainings to have robust enduring effects on behavior.

## Methods

### Overview.

We conducted our field experiment at a large global organization. Before the start of the experiment, the Institutional Review Board at the University of Pennsylvania reviewed and approved our study. Our partner organization emailed and invited 10,983 salaried employees to complete a new inclusive leadership workplace training that had been developed by the organization in partnership with a university. The training was not mandatory, but the partner organization strongly encouraged its employees to participate. The organization conveyed the value and importance of completing the training through an email campaign and also defaulted eligible employees into a scheduled appointment to complete it.

### Procedures.

Employees who consented to participate in our study were asked to complete a roughly hour-long training program (median completion time = 68 min) that took place entirely online. During the consent process, participants provided their email addresses and phone numbers, allowing for follow-up communication from the research team. Participants were able to stop the training at any time.

Immediately after consenting to participate, employees were randomized into one of three experimental conditions: a gender-bias training condition, a general-bias training condition, or an active placebo control condition. In the gender-bias training condition, all materials focused on explaining *gender* bias and *gender* stereotyping. In the general-bias training condition, participants were provided with information about bias and stereotypes relating to gender, race, age, sexual orientation, and obesity. These two conditions were completely identical except for the fact that we named different social categories at various points in the two conditions (e.g., in the gender-bias condition, we asked participants to estimate the percent of Fortune 500 CEOs who are women; in the general-bias condition, we asked participants to estimate the percent of Fortune 500 CEOs who are black). We observed minimal differences between the bias training that mentioned only gender and the bias training that mentioned gender, race, age, sexual orientation, and obesity, so for our primary analyses reported in the paper, we collapsed these two treatment conditions into a single “treatment condition.” Directionally, the gender-bias training appeared to be more effective than the general-bias training, but these results were not consistent (see *SI Appendix*, Tables S1–S3 for comparisons between the two treatment conditions). Results split apart comparing the gender bias to the control and the general bias to the control are available in *SI Appendix*, Tables S4–S9.

The content of the training in the treatment condition was divided into five sections. The first section (“What are [gender] stereotypes and why do they matter?”) introduced the basic psychological processes that underlie stereotyping, explained what stereotypes are, and discussed how stereotypes can result in bias (both conscious and unconscious) and undesired outcomes in the workplace. The word “gender” was omitted from this title and all subsequent titles in the general-bias training condition. The second section (“How do [gender] stereotypes apply at work?”) presented research to illustrate how bias and stereotyping manifest in the workplace. In the third section (“A test of associations”), participants were provided with personalized feedback about their own potential implicit biases by completing the Gender-Career Implicit Associations Test (18, 31). The fourth section (“How can we overcome [gender] stereotypes?”) taught participants strategies they can use to overcome stereotypes and biases in the workplace and provided participants with the opportunity to practice using those strategies. The fifth section (“Survey questions and feedback”) contained our attitude measures. Screenshots of the training are available in the *SI Appendix*.

The training employees received in the control condition contained content that was relevant to the workplace and could reasonably be labeled an inclusive leadership training, but it did not mention bias or stereotyping. Specifically, employees in the control condition learned about psychological safety and active listening. The components of the control condition matched the length and feel of the training in the treatment condition—both trainings contained videos, anecdotes, interactive questions, open-ended responses, surveys, strategies, and deliberate practice in the same formats—but with different content. The five sections of the control training were entitled “Why is inclusive leadership important?,” “What makes teams more inclusive?,” “What strategies can you use to build psychological safety?,” “A test of listening skills,” and “Survey questions and feedback.” Screenshots of the control training are available in the *SI Appendix*.

One week after the recruitment period for the training had ended, all employees across conditions received an email asking them to complete a voluntary follow-up survey to help address inequalities that women and racial minorities face in the workplace. In addition, we texted all employees roughly once a week for 12 wk after they completed the training. The content of these texts was the same across conditions (e.g., “Have you used any inclusive leadership strategies this week? Respond Y for Yes or N for No. Text STOP to unsubscribe”; “Is there a dark side to hiring for cultural fit? [web_link] Reply STOP to unsubscribe.”) Employees were able to opt out of the texts at any time.

### Attitude Measures.

At the conclusion of the training, we included a series of questions to measure employees’ attitudes. Because attitudes were measured at the end of training, our sample for these measures includes only those who reached this point in the training. Because additional attrition occurred at each measure, 77.3% of participants completed the first attitude measure, 77.2% completed the second, and 75.7% completed the third.

#### Attitudinal support for women.

We adapted a validated and widely used scale (21) to assess whether our intervention had a positive effect on attitudes relating to women and to measure the attitudes held by different demographic subgroups (based on their average responses to this scale in the control condition). This scale measures disagreement with claims about continued discrimination against women, a lack of support for women’s demands, and a lack of support for policies designed to help women. The scale asks participants to rate their level of agreement with statements, such as “Discrimination against women is no longer a problem” and “Society has reached the point where women and men have equal opportunities for achievement” on a scale from −3 (Strongly Disagree) to 3 (Strongly Agree). We adapted the scale to remove references to the United States since our participants came from many countries across the world. In addition, for interpretability of results, we coded the scale so that higher scores reflect more supportive attitudes toward women (i.e., more support for policies designed to help women and more recognition of discrimination against women). See *SI Appendix* for the exact items used. This was the second measure collected at the conclusion of the training, and 77.2% of participants who began the training completed it.

#### Gender bias acknowledgment.

We also measured people’s perceptions of their own bias and their perceptions of other people’s biases. These self-other measures were designed to capture people’s willingness to acknowledge the extent to which their personal biases matched those of the general population, following previous work on the bias blind spot (22, 32). The questions we asked were as follows: “To what extent do you believe that you exhibit gender stereotyping?” and “To what extent do you believe that the average person exhibits gender stereotyping?” Participants responded to each question on a scale from 1 (Not at all) to 7 (Very Much). We calculated the difference between these two items to measure participants’ willingness to acknowledge that their own gender biases may match those of the general population. This was the first measure collected at the conclusion of the training, and 77.3% of participants who began the training completed it.

#### Racial bias acknowledgment.

We also asked participants questions about perceptions of their own bias and their perceptions of other people’s biases with regards to racial stereotyping. The questions we asked were as follows: “To what extent do you believe that you exhibit racial stereotyping?” and “To what extent do you believe that the average person exhibits racial stereotyping?” Participants responded to each question on a scale from 1 (Not at all) to 7 (Very Much). We again calculated the difference between these two items to measure participants’ willingness to acknowledge that their own racial biases may match those of the general population.

#### Gender inclusive intentions.

We created a situational judgment test to assess behavioral intentions in situations where bias or stereotyping may arise. Participants were presented with different scenarios that can occur in the workplace. From a list of options, they then selected how they intended to behave in a given scenario. The situations and response options were created based on interviews with employees at our partner organization to provide construct validity. We used this measure to assess whether participants chose behaviors that would promote inclusivity in different situations that commonly arise in the workplace.

For each scenario, participants were asked to select which option they would be most likely to pursue and which option they would be least likely to pursue. Each scenario had one option that was particularly effective at promoting inclusion and one option that encouraged bias or stereotyping. Participants received one point for choosing the option that promoted inclusion as their most likely choice; received one point for choosing the option that encouraged bias as their least likely choice; lost one point for choosing the option that promoted inclusion as their least likely choice; and lost one point for choosing the option that encouraged bias as their most likely choice. Each scenario could thus be scored from −2 to 2. There were 10 scenarios in the measure, so total scores could range from −20 to 20 where higher scores reflect greater intentions to promote inclusivity in the workplace. In a separate sample, we validated that this measure is correlated with behavior that promotes gender inclusivity even after controlling for explicit and implicit attitudes (see *SI Appendix* for the exact items used and additional details on this validation). This was the third measure collected at the conclusion of the training, and 75.7% of participants who began the training completed it.

### Behavioral Measures.

We also examined whether the intervention promoted inclusive behaviors toward women and racial minorities in the workplace. We unobtrusively observed real workplace behaviors in programs created by our partner organization for the purposes of this study. These programs were administered up to 20-wk post-training to assess whether our intervention produced any lasting effects on behavior (see *SI Appendix*, Fig. S1 for a study timeline). These programs had no explicit or ostensible connection to the intervention, reducing the possibility that demand effects, self-presentation concerns, or social desirability concerns drove participant behavior. When analyzing our behavioral measures, we employed an intention-to-treat strategy and analyzed data from all 3,016 employees who consented to participate in the experiment regardless of whether they completed the training they were assigned to take online.

#### Connectivity and informal mentoring* program.

Three weeks after the recruitment period for the inclusive leadership training concluded, our partner organization sent everyone invited to the training an email describing a new program the organization had established. Specifically, the email described a new initiative to strengthen connections among colleagues and foster inclusiveness in the workplace, and employees were offered the chance to nominate up to five colleagues to informally connect with and mentor over coffee through this program. Gift certificates for coffee were provided to everyone who signed up to facilitate mentoring meetings. This was a real program, and it had no explicit or ostensible connections to our intervention. The behavioral measure of interest to us was the number of women selected to be informally mentored by each consented participant (participants who chose not to participate in the program were counted as nominating zero women). In the United States, we were also able to measure the number of racial minorities selected to be informally mentored by each consented participant in each condition.

#### Recognition for excellence program.

Six weeks after the recruitment period for our inclusive leadership training had ended, our partner organization sent everyone invited to the training an email soliciting nominations to recognize a colleague’s excellence. The behavioral measure of interest was the number of women nominated by each consented participant. In the United States, we were also able to measure the number of racial minorities nominated by each consented participant in each condition (participants who chose not to participate in the program were counted as nominating zero women and zero racial minorities).

#### Willingness to talk to a female versus male new hire audit experiment.

Fourteen weeks after the recruitment period for our inclusive leadership training concluded, our partner organization emailed everyone invited to the training asking if they would be willing to speak to either a male new hire or a female new hire (randomly assigned) in an audit study design (23, 33). We measured the difference in the percent of consented study participants who volunteered to speak to the male versus the female new hire by condition to assess levels of gender bias by experimental condition. There was a typographical error in one version of the emails that affected 1,098 out of 2,898 (37.9%) of the emails sent. Although the emails described speaking to a male new hire and generally used male pronouns, there was one instance of referring to the new hire as a “she” in the middle of the emails. Removing these data from our analyses does not significantly alter our results and, in fact, increases our estimate of the treatment effect.

## Supplementary Material

Supplementary File

## Footnotes

*We use the word mentorship for simplicity and ease of understanding in our description. The program did not explicitly use the word mentorship but instead focused on connectivity to avoid confusion or conflicts with other formal mentorship programs offered by our field partner.
