# Supplementary material for: The mixed effects of online diversity training
Source: Proc Natl Acad Sci U S A. 2019 Apr 1;116(16):7778–83. doi: 10.1073/pnas.1816076116 (PMC6475398; doi:10.1073/pnas.1816076116)
Supplement: Supplementary File [file pnas.1816076116.sapp.pdf]

Supporting Information (SI) for

"The Mixed Effects of Online Diversity Training"

This file includes:

Summary Statistics
- Study Population pg. 2
- Summary of Attitudes Towards Women in the Absence of Intervention pg. 2

Data Validations
- Validation of Random Assignment pg. 5
- Ruling Out Response Biases for Behavioral Measures pg. 5

Results
- Analysis Strategy pg. 7
- Attitudes Pertaining to Women pg. 8
- Behaviors Pertaining to Women pg. 11
- Moderation of Gender Results by Attitudes in the Absence of Intervention pg. 14
- Attitudes Pertaining to Racial Minorities pg. 18
- Behaviors Pertaining to Racial Minorities pg. 19
- Additional Robustness Checks pg. 21

Survey Instruments and Instrument Validation
- Attitudinal Support for Women pg. 23
- Gender Bias Acknowledgment pg. 23
- Racial Bias Acknowledgment pg. 23
- Gender Inclusive Intentions pg. 23
- Validation of Situational Judgment Test for Gender Inclusive Intentions pg. 28

Figs. S1 to S3 pg. 36
Tables S1 to S28 pg. 39
Screenshots of Intervention pg. 69
References pg. 78

Link to Data and Code: https://osf.io/drz2j/?view_only=4c5c8963550e469f86755f2000f1d6e2

SUMMARY STATISTICS

Study Population

We conducted our field experiment at a large global organization. The organization we partnered with has offices on every continent except Antarctica. The organization co-developed and deployed our training as part of a broader strategic effort around inclusion and inclusive leadership training.

We recruited as many employees as possible at the firm to participate in the study over the course of six weeks. Of the 10,983 employees who received an email with an invitation to take the training, 3,016 (27.5%) started the training and consented to participate in our experiment. Of the employees who started the training, 2,282 (75.7%) completed the hour-long training. Our entire sample of 3,016 study participants was 61.5% male and included employees located in 63 different countries (38.5% were located in the United States). In the U.S., 56.3% of participants were male, while 64.7% of participants outside of the U.S. were male. In the U.S., 59.4% of participants were white (our partner organization does not track race of employees outside of the U.S.). The median completion time of the training was 68 minutes. The length of the training was selected not because we thought there was something special about one hour of education but instead in response to various considerations at the organization we partnered with, including employee bandwidth. Additional details including balance checks to ensure the success of balanced random assignment to conditions are available in the section *Data Validations: Validation of Random Assignment.*

Summary of Attitudes Towards Women in the Absence of Intervention

In our pre-analysis plan, we pre-registered that we would examine our data by two demographic splits (see https://www.socialscienceregistry.org/trials/2200). Specifically, we pre-

registered that we would analyze our participants by gender (men vs. women) and by location (in the U.S. vs. outside the U.S.). Past surveys have shown that women generally have more supportive attitudes towards women in the workplace and towards feminist issues than do men (Davis & Robinson, 1991; Inglehart & Norris, 2003), so we expected that women's attitudes would be particularly supportive of women relative to men's attitudes. Similarly, past surveys have shown that attitudes in the United States are more progressive towards gender equality than internationally (Brandt, 2011)–though there is, of course, considerable heterogeneity within the U.S. and across and within other countries–so we expected the attitudes of employees in the U.S. to be relatively progressive on gender, and somewhat more so than those of employees outside the U.S.

We examined the attitudinal support for women among participants in our control condition in order to provide a measure of the attitudes held by different subgroups in the absence of a diversity training intervention. We used the attitudinal support for women scale to verify these predicted differences because it is a minor adaptation of a scale that has been previously validated extensively in the literature (Swim, Aikin, Hall, & Hunter, 1995).

As anticipated, we found that among study participants who were not exposed to our treatment intervention (that is, participants in our control group), women exhibited higher average standardized[1] levels of attitudinal support for women than men ($M_{women} = 0.269$, $SD_{women} = 0.92$; $M_{men} = -0.346$, $SD_{men} = 1.12$; $t(781) = 8.03$, $p < 0.0001$). In addition, we found that employees located in the U.S. exhibited higher average standardized levels of attitudinal support

[1] Unstandardized means and standard deviations for this measure are not reported due to confidentiality requirements of our partner organization. However, it is worth noting that none of the subgroups exhibited average scores that fell below the midpoint of the scale measuring attitudinal support for women.

for women than employees outside the U.S. ($M_{U.S.} = 0.288$, $SD_{U.S.} = 1.03$; $M_{international} = -0.358$, $SD_{international} = 1.05$; $t(781) = 8.47$, $p < 0.0001$).

In exploratory analyses, we examined the intersection of these two demographic splits. We found that women in the U.S. exhibited the highest average levels of attitudinal support for women (see Figure S2), men in the U.S. and women outside the U.S. exhibited slightly less attitudinal support for women (and these two groups did not differ significantly from one another), and finally, men outside the U.S. who did not experience our diversity training exhibited somewhat lower average levels of attitudinal support for women than these other groups (see Figure S2).

DATA VALIDATIONS

Validation of Random Assignment

We found no evidence of differential attrition between our treatment and control conditions. The training completion rate in the control condition was 75.1%, while the completion rate in the treatment condition was 75.9% ($z = 0.467$; $p = 0.640$). Our sample was also balanced in terms of demographics (see Table S10), indicating that randomization was successful. Employees rated the control training as more valuable ($M = 5.27$; $SD = 1.33$) than the treatment training ($M = 4.93$; $SD = 1.39$; $t(2278) = 5.65$, $p < 0.0001$) and spent more time on the control training than the treatment training (median completion time for control = 70.7 minutes; median completion time for treatment = 65.5 minutes). This might suggest that, if anything, demand effects would bias employees towards greater responsiveness to follow-up measures in the control condition rather than the treatment condition.

Ruling Out Response Biases for Behavioral Measures

In addition to collecting measures of actual workplace behaviors in programs that were in no ostensible way connected to our diversity training intervention, we collected two follow-up measures that were explicitly connected to the inclusive leadership training, as described in the *Procedures* section. These measures were delivered by our research team and were labeled as follow-ups to the training, allowing us to test whether the response rates differed between treatment and control (note that this concern is also mitigated by the fact that we use an intent-to-treat framework for analyzing our measures of real workplace behaviors).

First, we measured what proportion of employees in each condition clicked through to take a voluntary follow-up survey sent by the research team associated with the training. We found no differences in willingness to take this follow-up survey between conditions ($M_{treatment} =$

15.4%; $M_{control}$ = 15.7%; z = 0.278, p = 0.781). Second, we measured the average number of engagements with text messages per employee across conditions where engagements were defined as any reply to a text or click-through to a linked article or video. We found no differences in engagement with text messages from the research team across conditions ($M_{treatment}$ = 0.507 engagements, $SD_{treatment}$ = 1.16 engagements; $M_{control}$ = 0.549 engagements, $SD_{control}$ = 1.25 engagements; t(3,014) = 0.891, p = 0.373).

The fact that we found no differences in response rates to these two follow-ups explicitly connected to our intervention and that attrition was also nearly identical in our treatment and control groups suggests that our experimental conditions were successfully balanced in the degree of engagement they produced.

RESULTS

Analysis Strategy

We compared participants across conditions on each outcome measure using t-tests and ordinary least squares (OLS) regressions, following our pre-analysis plan. Our pre-analysis plan was uploaded to the AEA RCT Registry (https://www.socialscienceregistry.org/trials/2200) after recruitment for the study started but before any data were received from our field partner. First, we conducted pairwise t-tests between treatment and control for each of our dependent variables about attitudes and behaviors pertaining to women[2] (see Tables S11, S12). Next, we ran regressions to see the effects of the treatment condition on these dependent variables in models that include controls for each subgroup (see Tables S13, S14). Finally, we present results from regressions that include interaction terms to see how the intervention varies in effectiveness between pre-registered subgroups (i.e., men and women; participants based in the United States and those based outside of the United States; see Tables S15-S17). Our pre-analysis plan did not specify the exact functional form of our regression models because we did not know in advance what demographic variables our field partner would be able to provide us, so we use all available participant-invariant characteristics as controls (namely: office location, job category, race, and gender). Specifically, our standard ordinary least squares regression with controls predicts each outcome using all interactions between (a) an indicator for being in the treatment condition, (b) an indicator for being male, and (c) an indicator for being located in the U.S., as well as fixed effects for race, exact office location, and job category. Following our pre-analysis plan, for outcome measures that require nominating colleagues, we also cluster standard errors by office

[2] This is true for all dependent variables except for the audit study, where the outcome of interest is the difference in the willingness to talk to the female new hire relative to the male new hire. Thus, for the audit study only, we are interested in the interaction between the gender of the new hire and condition.

to account for the fact that employees are likely to nominate colleagues who work in the same office as them. For ease of interpretability, all attitudinal outcome measures have been standardized.

Results of our intervention's effects on attitudes and behaviors pertaining to racial minorities are available in Tables S18-S20. For these results, we focus our attention on employees in the U.S., given that our field partner only collects data on the race of its employees in the U.S. We also break down our results pertaining to racial minorities by white employees and racial minority employees in the U.S., just as we break down results involving attitudes and behaviors pertaining to women between men and women inside and outside of the U.S.

Attitudes Pertaining to Women

Note that whenever we report on attitudinal variables, we standardize the variable to report its level in z-scores.

Attitudinal Support for Women. Employees in the treatment condition exhibited significantly higher levels of attitudinal support for women than employees in the control condition post-intervention ($M_{treatment} = 0.055$, $SD_{treatment} = 0.95$; $M_{control} = -0.108$, $SD_{control} = 1.09$; $t(2325) = 3.736$, $p = 0.0002$, $d = 0.164$; see Table S11). This difference suggests that our treatment had a significant, positive effect on employees' attitudes towards women. However, the overall positive treatment effect was largely driven by differences among employees outside the U.S.: there were significant differences between the treatment and control conditions for men outside the U.S. ($M_{treatment} = -0.264$, $SD_{treatment} = 0.94$; $M_{control} = -0.516$, $SD_{control} = 1.08$; $t(891) = 3.618$, $p = 0.0003$, $d = 0.253$) and women outside the U.S. ($M_{treatment} = 0.198$, $SD_{treatment} = 0.88$; $M_{control} = -0.047$, $SD_{control} = 0.89$; $t(499) = 2.91$, $p = 0.0038$, $d = 0.278$), but there were no significant differences for men in the U.S. ($M_{treatment} = 0.080$, $SD_{treatment} = 0.95$; $M_{control} = -0.011$,

$SD_{control}$ = 1.11; t(524) = 0.966, p = 0.335) or women in the U.S. ($M_{treatment}$ = 0.526, $SD_{treatment}$ = 0.80; $M_{control}$ = 0.631, $SD_{control}$ = 0.81; t(405) = 1.256, p = 0.210).

We ran our standard ordinary least squares (OLS) regression predicting standardized attitudinal support for women where we included fixed effects for an employee's office location, job category, race, and all interactions between (a) the treatment condition, (b) an indicator for being male, and (c) an indicator for being located in the U.S. (see Table S15, Model 2). We found a significant main effect of the treatment (b = 0.149, p < 0.001)[3], suggesting that our diversity training intervention had a significant positive effect on attitudes. Further, we observed a significant interaction between the treatment and an indicator for being located in the U.S. (b = -0.333, p = 0.012), indicating that the treatment had a larger positive effect on the attitudes of employees outside the U.S. than employees in the U.S.

Gender Bias Acknowledgment. Employees in the treatment condition were more willing to acknowledge that their own gender biases matched those of the general population compared to employees in the control condition ($M_{treatment}$ = 0.077, $SD_{treatment}$ = 0.97; $M_{control}$ = -0.128, $SD_{control}$ = 1.04; t(2330) = 4.69, p < 0.0001, d = 0.205; see Table S11). The increase in acknowledgment was driven by significantly higher perceptions of employees' own gender bias and stereotyping in the treatment condition compared to the control condition ($M_{treatment}$ = 0.193, $SD_{treatment}$ = 0.96; $M_{control}$ = -0.381, $SD_{control}$ = 0.97; t(2333) = 13.60, p < 0.0001; d = 0.596). Perceptions of others' gender bias and stereotyping were also significantly higher in the treatment condition than in the control condition ($M_{treatment}$ = 0.158, $SD_{treatment}$ = 0.92; $M_{control}$ = -0.312, $SD_{control}$ = 1.08; t(2333) = 10.98, p < 0.0001, d = 0.491). All pre-registered demographic

[3] For all treatment effect estimates derived from our regressions, we use our standard pre-registered ordinary least squares regression specification with all controls. To calculate treatment effects for specific subgroups, we use Wald tests to sum the relevant treatment effect coefficients in the correct proportions.

subgroups showed significant differences between the treatment and control conditions (see Table S11).

In our standard pre-registered ordinary least squares regression with all controls, we still observed a significant main effect of the treatment condition ($b = 0.217$, $p < 0.001$; see Table S15, Model 4). We did not find any significant interactions between the treatment and the demographic subgroups of interest (employees in the U.S. and male employees).

Gender Inclusive Intentions. Employees in the treatment condition earned significantly higher scores on the situational judgment test measuring gender-inclusive intentions than employees in the control condition ($M_{treatment} = 0.042$, $SD_{treatment} = 1.04$; $M_{control} = -0.084$, $SD_{control} = 0.90$; $t(2280) = 2.85$, $p = 0.0044$, $d = 0.13$; see Table S11). The overall treatment effect was largely driven by intention change in employees located outside of the U.S.: there were significant differences between the treatment and control conditions for men outside of the U.S. ($M_{treatment} = -0.065$, $SD_{treatment} = 1.03$; $M_{control} = -0.222$, $SD_{control} = 0.89$; $t(867) = 2.26$, $p = 0.024$, $d = 0.16$) and marginally positive effects for women outside of the U.S. ($M_{treatment} = 0.097$, $SD_{treatment} = 1.01$; $M_{control} = -0.087$, $SD_{control} = 0.91$; $t(489) = 1.93$, $p = 0.054$, $d = 0.19$), but there were no significant differences for men in the U.S. ($M_{treatment} = 0.032$, $SD_{treatment} = 1.03$; $M_{control} = -0.028$, $SD_{control} = 0.95$; $t(519) = 0.63$, $p = 0.53$) or women in the U.S. ($M_{treatment} = 0.214$, $SD_{treatment} = 1.10$; $M_{control} = 0.161$, $SD_{control} = 0.82$; $t(399) = 0.49$, $p = 0.62$; see Table S11).

We also ran our standard pre-registered ordinary least squares regression with all controls to predict scores on this measure. We again saw a significant main effect of the treatment ($b = 0.147$, $p = 0.001$; see Table S15, Model 6), but we observed no significant interactions.

Behaviors Pertaining to Women

Informal Mentoring[4] of Women (~3 Weeks After Recruitment Ended). We found no overall differences between our treatment and control groups on the average number of women selected for informal mentorship per consented participant ($M_{treatment}$ = 0.154, $SD_{treatment}$ = 0.66; $M_{control}$ = 0.147, $SD_{control}$ = 0.64; t(3014) = 0.278; p = 0.781). However, when we decomposed the treatment effect by demographic subgroup, we found that U.S. women selected significantly more women in the treatment condition than in the control condition (average number of women selected by U.S. $women_{treatment}$ = 0.27, $SD_{treatment}$ = 0.88; average number of women selected by U.S. $women_{control}$ = 0.073, $SD_{control}$ = 0.46; t(504) = 2.775, p = 0.0057; see Table S12). This difference remains significant when we operationalize our dependent variable as a binary outcome measuring whether an employee selected any women at all (any women selected by U.S $women_{treatment}$ = 10.4%, any women selected by U.S. $women_{control}$ = 3.35%, z = 2.81, p = 0.0050).

We also ran our standard pre-registered ordinary least squares regression with all controls with clustered standard errors by office location predicting the number of women selected per employee. We found a significant interaction between the treatment condition and an indicator for being located in the U.S. (b = 0.292, p = 0.003) as well as a significant three-way interaction between the treatment condition, an indicator for being male, and an indicator for being located in the U.S. (b = -0.243, p = 0.015; see Table S16, Model 2), suggesting that women in the U.S. were most likely to change their behaviors in response to the intervention. In terms of real-world significance, these estimates suggest that for every five women in the U.S. who were in the treatment condition (instead of the control), an additional woman was selected through this

[4] We use the word "mentorship" for simplicity and ease of understanding. The program did not explicitly use the word "mentorship" but instead focused on "connectivity" to avoid confusion or conflicts with other formal mentorship programs offered by our field partner.

program (a Wald test adding together the coefficients for *Treatment* and *Treatment* x *Male Employee* provides a treatment effect estimate for women in the U.S. of b = 0.203, p = 0.001).

In exploratory analyses suggested by our organizational partner, we examined tenure differences between the employees who participated in our study and those they invited to meet for coffee in the informal mentoring program. While this program was designed so employees could volunteer to provide informal mentoring to *others*, we discovered that many participants elected to invite employees who were senior to them in the organization to meet for coffee, suggesting that many were using this program to seek out mentorship, rather than provide mentorship. To determine if an employee was seeking out mentorship or providing mentorship, we used data provided by our field partner. If an employee selected someone who was either a) at a higher level in the organization, as determined by our field partner, or b) at the same level in the organization but had spent more years at the organization, then we categorized that selection as "seeking mentorship," rather than "providing mentorship." If an employee selected someone who was either 1) at a lower level in the organization or 2) at the same level in the organization but had spent fewer years at the organization, then we categorized that selection as "providing mentorship."

We then re-ran our standard pre-registered ordinary least squares regression with these two new outcome variables ("providing mentorship" and "seeking mentorship") and clustered standard errors by office location. Specifically, one outcome variable was the number of women invited to coffee through this mentoring program who were senior to the employee in our study and the other was the number of women invited to coffee through this mentorship program who were junior to the employee in our study. We see the same pattern of results for both measures, although the results appear driven by women in the U.S. seeking out more mentoring: women in

the U.S. aimed to provide marginally more mentorship to women in the treatment condition relative to the control condition ($b = 0.0568$, $p = 0.067$) in addition to seeking out more informal mentorship from other women ($b = 0.141$, $p = 0.001$; see Table S21). More generally, women in the U.S. in the treatment condition sought out more mentorship from more senior colleagues regardless of gender ($b = 0.221$, $p < 0.001$), suggesting that our intervention prompted women in the U.S. to take more initiative in overcoming any potential obstacles or barriers they face in the workplace.

Recognition of Women for Excellence (~6 weeks after recruitment ended). We found no differences in the number of women recognized for excellence per consented participant across conditions ($M_{treatment} = 0.0155$, $SD_{treatment} = 0.127$; $M_{control} = 0.0119$, $SD_{control} = 0.108$; $t(3014) = 0.763$; $p = 0.446$; see Table S12). Using our standard pre-registered regression model (see Table S16), we found a marginal positive treatment effect on the number of women nominated for excellence awards by employees in our study who worked in the U.S. ($b = 0.012$, $p = 0.075$).

Willingness to Talk to a Female versus Male New Hire Audit (~14 weeks after recruitment ended). We found no significant differences in employees' willingness to speak to a female or male new hire when we compare the treatment and control conditions (percent willing to talk to $\text{female}_{treatment} = 42.3\%$; percent willing to talk to $\text{female}_{control} = 39.1\%$; percent willing to talk to $\text{male}_{treatment} = 34.8\%$; percent willing to talk to $\text{male}_{control} = 35.1\%$; difference in percent willing to talk to female vs. $\text{male}_{treatment} = 7.44\%$; difference in percent willing to talk to female vs. $\text{male}_{control} = 3.99\%$; $F(1, 2894) = 0.82$, $p = 0.366$; see Table S12). Notably, we found that participants in the treatment condition were significantly more willing to talk to a female new hire than a male new hire ($p = 0.0008$). Collapsing across female employees and using our

standard pre-registered regression model to estimate treatment effects (see Table S17), we found that the intervention did have a significant effect among female employees, leading them to favor speaking with a female new hire over a male new hire (b = 0.127; P = 0.047).

Moderation of Gender Results by Attitudes in the Absence of Intervention

In exploratory analyses, we tested for additional evidence to support our proposed model of behavior change. In particular, we tested whether those whose untreated attitudes were more aligned with our intervention would change their behaviors more and their attitudes less in response to our intervention, while those whose untreated attitudes were less aligned with our intervention would change their attitudes more and their behaviors less in response to our intervention. We do not know the pre-treatment attitudes of participants because they were not measured to avoid demand and anchoring effects (Orne, 1962; Tversky & Kahneman, 1974; Zizzo, 2010). However, we can examine the attitudes of participants in the control condition in different subgroups as a proxy for pre-treatment attitudes.

We created participant subgroups by both gender and exact country of residence (a narrower category than our pre-registered U.S. vs. international classification). This allows us to analyze 86 country-gender subgroups, with an average of 34.1 participants per subgroup.[5] We can then use the attitudinal support for women[6] exhibited by untreated participants in the subgroup of interest (e.g., women in France) as a proxy for where participants in that subgroup stood along an attitude continuum pre-training from most to least aligned with our training's

[5] Although we have 63 countries represented in our data overall, not all countries had men and women as participants or had participants in both the treatment and control conditions, which means we must exclude these country-gender pairs from our analysis.
[6] We use attitudinal support for women for all of these tests since it is the only attitude measure we collect which is based on a scale that has been previously validated extensively in the literature (Swim, Aikin, Hall, & Hunter, 1995).

message.[7] The standard deviation in the attitudinal support for women scores across these 86 subgroups was 0.541, and the absolute difference between the subgroups with the highest and lowest average scores was 3.25.

The Intervention's Impact on Attitudes. We used our proxy measure of pre-training attitudinal support for women in moderation analyses. It is important to note that, on average, employees in our study had attitudes well above the midpoint of our scale. We tested whether a subgroup's pre-training attitudes predicted which subgroups' attitudes shifted the most in a series of regressions. To test this, we ran an ordinary least squares regression predicting attitudes after training with an interaction between an indicator for the treatment condition and a continuous standardized variable for our proxy of pre-training attitudes. When predicting our intervention's impact on attitudinal support for women, we found a significant interaction between the treatment and an employee's subgroup's pre-training attitudes ($b = -0.273$, $p < 0.001$; see Table S22, Model 2). Specifically, those who showed the most movement were those whose attitudes in the absence of intervention–while still supportive of women–were less so than those of other employees. In charts depicting the relationship between a subgroup's pre-training attitudes and its subsequent attitudinal shift as a result of the intervention, we found the same pattern of results (see Figure S3, Panel A). Similarly, when predicting scores on our situational judgment test, we found significant interactions in the predicted direction between the treatment and an employee's subgroup's pre-training attitudes ($b = -0.121$, $p = 0.023$, see Table S22, Model 6). While employees' perceptions of other people's gender bias showed the expected interaction, this was not true for perceptions of their own gender bias, so we did not find the expected interaction

[7] This proxy measure is obviously an imprecise way to measure pre-treatment attitudes, which means we have measurement error. This should not introduce bias into our estimates unless we believe that the measurement error is correlated with the true value, which we have no reason to expect. Measurement error does, however, mean that our standard errors increase, making it harder for us to detect significant effects.

when predicting the size of the gap between an employee's perceptions of their own gender bias and their perceptions of other people's gender bias.

The Intervention's Impact on Behaviors. Using the same proxy measure for our finer-grained subgroups' pre-training attitudes, we tested whether a subgroup's pre-training attitudes predicted its degree of behavior change in response to our intervention. Specifically, we ran a series of OLS regressions (see Table S22) predicting behaviors after training with a treatment indicator variable, a continuous standardized variable for our proxy of pre-training attitudes (i.e. a demographic subgroup's average level of attitudinal support for women in the control condition), and–as our predictor of primary interest–an interaction between an indicator for the treatment condition and the continuous standardized variable for our proxy of pre-training attitudes. For the informal mentoring program and the award nomination program, we cluster standard errors by office location to account for the fact that employees are likely to select people with whom they share an office location. In the audit study, our dependent variable is the difference in the percent of employees willing to talk to a female new hire relative to a male new hire by condition. Hence, we need to interact these willingness-to-talk terms with an indicator for whether the new hire was female to determine if our treatment had the predicted effect.

We found some evidence suggesting stronger behavioral effects of the intervention among subgroups whose untreated attitudes were more aligned with our intervention's message. Specifically, as shown in Table S22, Model 8, there was a significant interaction between receiving our intervention and an employee's subgroup's pre-training attitude alignment when predicting the number of women an employee chose to informally mentor ($b = 0.112$, $p = 0.012$). The largest effects on behaviors occurred for those subgroups whose attitudes were most supportive of women (see Figure S3, Panel B). To put the magnitude of this effect in context, our

results suggest that an additional woman received informal mentoring as a result of our intervention for every twenty employees trained in the subgroup whose attitudes were least aligned with our intervention, while an additional woman received informal mentoring as a result of our intervention for every five employees trained in the subgroup whose attitudes were most aligned with our intervention. We did not, however, find significant interactions between pre-training attitudes and our treatment condition when predicting other behavioral measures.

Moderation Robustness Checks. In addition to our moderation analyses using country-gender subgroups, we also ran moderation analyses using finer-grained office location-gender subgroups (note that there is often more than one office location in a country). Using the same method discussed above, we used attitudinal support in the control group as a proxy for pre-training attitudes in each subgroup. We were able to create 159 office location-gender subgroups with an average of 17.9 employees per subgroup. We then repeated each of our moderation analyses. The results of these analyses are listed in Table S23 and are extremely similar to those that rely on country-gender subgroups, providing further support for our proposed model of behavior change. In particular, we found evidence suggesting that the treatment was more effective at increasing attitudinal support for women ($p < 0.001$; see Table S23, Model 2) and gender inclusive intentions ($p = 0.035$; see Table S23, Model 6) for employees whose pre-training attitudes were relatively less supportive of women. On the other hand, we found evidence that the treatment was more effective at increasing the number of women selected for informal mentoring[8] ($p = 0.037$; see Table S23, Model 8) for employees whose pre-training attitudes were relatively more supportive of women.

[8] Again, we use the word "mentorship" for simplicity and ease of understanding. The program did not explicitly use the word "mentorship" but instead focused on "connectivity" to avoid confusion or conflicts with other formal mentorship programs offered by our field partner.

We also used these office location-gender subgroups to examine variation within the United States to see if our proposed model of behavior change was supported within the country with the most participants in our sample. Limiting our data to participants in the United States left us with 43 office location-gender subgroups with an average of 26.3 employees each. We reran our moderation analyses using these U.S. office location-gender subgroups. The results of these analyses are listed in Table S24. We again found some evidence in support of our proposed model of behavior change. First, the treatment was more effective at increasing attitudinal support for women among employees whose pre-training attitudes were relatively less supportive of women ($p < 0.001$; see Table S24, Model 2). Second, the treatment was more effective at increasing willingness to talk to the female new hire relative to the mail new hire for employees whose pre-training attitudes were relatively more supportive of women ($p = 0.045$; see Table S24, Model 12).

Attitudes Pertaining to Racial Minorities

The one measure of attitudes pertaining to racial minorities that we collected was a racial bias acknowledgment measure (see section *Materials and Methods: Attitude Measures* for a definition). Employees in the treatment condition had a significantly smaller gap in their perceptions of racial bias and stereotyping by others versus themselves than did employees in the control condition ($t(2330) = 4.27$, $p < 0.0001$, $d = 0.187$; see Table S18). The smaller difference in self-other perceptions was driven by significantly higher perceptions of employees' own racial bias and stereotyping in the treatment condition compared to the control condition ($t(2330) = 14.04$, $p < 0.0001$; $d = 0.615$). Perceptions of others' racial bias and stereotyping were also significantly higher in the treatment condition than in the control condition ($t(2330) = 11.93$, $p < 0.0001$, $d = 0.522$). In our standard pre-registered OLS regression with all controls, we still

observed a significant main effect of the treatment condition ($b = 0.193$, $p < 0.001$). We did not find any significant interactions between the treatment and the demographic subgroups of interest (employees in the U.S. and male employees).

We also found evidence of positive spillover effects, as participants in our gender-focused intervention condition exhibited a significantly smaller gap in self-other perceptions of racial bias and stereotyping than did participants in our placebo control condition ($t(1575) = 2.66$, $p = 0.0079$; see Table S6). The smaller difference in self-other perceptions was driven by participants' significantly higher perceptions of their own racial bias and stereotyping in the gender-focused intervention condition compared to the control condition ($t(1575) = 12.04$, $p < 0.0001$). Perceptions of others' racial bias and stereotyping were also significantly higher in the gender-focused treatment condition than in the control condition ($t(1575) = 10.90$, $p < 0.0001$). Together, these results suggest that a diversity training focusing exclusively on gender bias and stereotyping can also have a positive effect on people's attitudes relating to racial minorities. The general-bias condition also had a significant positive effect on this measure ($t(1537) = 4.71$, $p < 0.001$; see Table S9).

Behaviors Pertaining to Racial Minorities

Because our field partner only collects data on the race of its employees in the United States, we can only analyze our behavioral measures when it comes to racial minorities for employees in the U.S.

A t-test comparing the average number of racial minorities selected for informal mentoring by U.S. participants exposed to our treatment condition and our control condition showed a directionally positive effect, whereby U.S. participants in the treatment condition chose to informally mentor marginally more racial minorities than participants in the control condition

($M_{treatment}$ = 0.123, $SD_{treatment}$ = 0.57; $M_{control}$ = 0.072, $SD_{control}$ = 0.40; t(1157) = 1.57, p = 0.116; see Table S18). We also ran an OLS regression predicting the number of racial minorities selected for informal mentoring using an indicator for the treatment condition, an indicator for the participant being white, the interaction between these two indicators, and fixed effects for office location, job category, race, and gender while clustering standard errors by office location (see Table S20). We again found a directionally positive effect of the treatment on the number of racial minorities selected for informal mentoring (b = 0.0470, p = 0.052).

A t-test comparing the average number of racial minorities recognized for excellence across conditions showed that U.S. participants in the treatment condition chose to recognize directionally more racial minorities than participants in the control condition ($M_{treatment}$ = 0.026, $SD_{treatment}$ = 0.17; $M_{control}$ = 0.0080, $SD_{control}$ = 0.089; t(1157) = 1.93, p = 0.054; see Table S18). We also ran an OLS regression with interactions and controls to predict the number of racial minorities recognized for excellence (see Table 20). We found a significant positive effect of the intervention on the number of racial minorities recognized for excellence (b = 0.0170, p = 0.039).

Interestingly, these effects on behaviors appeared to be directionally driven by the effects of the gender-bias treatment condition, as opposed to the general-bias treatment condition. Although there were no significant differences between these two conditions on these measures, an OLS regression showed a significant positive effect of the gender-bias treatment on the number of racial minorities selected for informal mentoring (b = 0.0539, p = 0.044), providing additional support for the existence of positive spillover effects of our training. Further, there was also a significant positive effect of the gender-bias intervention on the number of racial minorities recognized for excellence (b = 0.026, p = 0.016).

Additional Robustness Checks

In exploratory analyses encouraged by reviewers, we investigated whether there might be other factors that moderated the effects of our diversity training. For example, managers might have more incentives to attend to a diversity training because they may be evaluated in part based on their handling of diversity and inclusion.

Our organizational partner has two main types of employees. For one type of employee (73.9% of our sample), there is a strict hierarchy of ranks (or levels) in the organization, so we can easily identify who is a manager and who is not (25.1% of these employees are managers). For the second type of employee (the remaining 26.1% of our sample), there is not a clear hierarchy, and we only know how long each employee has been at the company. In this sub-sample, we are not able to easily classify who is a manager.

To test whether our training had different effects on managers as compared to other types of employees, we reran our primary analyses with the sub-sample of employees who could be classified as managers or not and included an interaction term between our treatment indicator and an indicator for whether a given employee was a manager. We do not see any evidence that the training was differentially effective for managers versus non-managers (all P's across all behavioral and attitudinal DVs > 0.1). We also reran these analyses interacting our treatment indicator with a continuous variable representing an employee's rank in the organization (again, these analyses necessarily only include the 73.9% of employees in roles with clearly defined ranks in our organizational partner's hierarchy). We find no evidence that the training was differentially effective for higher-ranked versus lower-ranked employees (again, all P's across all behavioral and attitudinal DVs > 0.1).

Given that the recruitment period for our training was six weeks long, we also examined whether there were differential effects of treatment depending on when an employee completed the training. Similar to the above analyses, we reran our primary analyses interacting our treatment indicator with a continuous variable representing the number of days from the start of the recruitment period to when an employee began the training. We find no evidence that the training was differentially effective depending on when an employee started the training (all P's across all behavioral and attitudinal DVs > 0.05).

Finally, we also tested whether our results differed if we analyzed only participants who completed the training, as compared to analyzing all participants who were randomized into a condition using our intention-to-treat analysis strategy. Interestingly, we do not find any significant differences in estimated treatment effects when limiting our analyses to only employees who completed the training in both conditions. This may be due to the fact that our training completion rate is relatively high (above 75%) in both conditions, and we do not observe any evidence of differential attrition by condition.

SURVEY INSTRUMENTS AND INSTRUMENT VALIDATION

Attitudinal Support for Women Scale (adapted from (Swim et al., 1995)**):**
Please rate the following items on a scale from -3 (Strongly Disagree) to 3 (Strongly Agree):

1. Discrimination against women is no longer a problem in society.
2. Women often miss out on good jobs due to sexual discrimination.
3. It is rare to see women treated in a sexist manner on television.
4. On average, people in our society treat husbands and wives equally.
5. Society has reached the point where women and men have equal opportunities for achievement.
6. It is easy to understand the anger of women's groups in society.
7. It is easy to understand why women's groups are still concerned about societal limitations of women's opportunities.
8. Over the past few years, the government and news media have been showing more concern about the treatment of women than is warranted by women's actual experiences.

Gender Bias Acknowledgment:

Gender Stereotyping: Many studies have found that we often make automatic assumptions about other people based on their gender. For example, people associate men with technology and women with housework.

Please answer the following questions on a scale from 1 (Not at all) to 7 (Very Much):

1. To what extent do you believe that you exhibit gender stereotyping?
2. To what extent do you believe that the average person exhibits gender stereotyping?

Racial Bias Acknowledgment:

Racial Stereotyping: Many studies have found that we often make automatic assumptions about other people based on their race. For example, people associate Asians with being good at math and Blacks with being athletic

Please answer the following questions on a scale from 1 (Not at all) to 7 (Very Much):

1. To what extent do you believe that you exhibit racial stereotyping?
2. To what extent do you believe that the average person exhibits racial stereotyping?

Gender Inclusive Intentions:

[NOTE: The words “project”, “organization”, and “junior colleagues” in the survey instrument printed below have replaced words that could potentially identify our organizational partner. We have also removed one additional word that could identify our organizational partner. All edits are embedded in brackets below.]

Instructions: For the following 10 questions, please read each scenario carefully and choose two responses:

1) the response that **most likely** reflects what you would do and
2) the response that **least likely** reflects what you would do.

Some questions may involve descriptions of situations you normally face, and some may not. Sometimes you might think of another strategy you might use. That is okay. Please choose from the responses presented.

1. Sara and Joe were members of a large team you were on last year. You think that they are both excellent data analysts, though you don't know either of them well personally. Both Sara and Joe have decided to apply to a fellowship, and both asked you to write a recommendation letter. There is only one fellowship available. What would you be most likely and least likely to do?

Least Likely				Most Likely	
(0)	☐	1.	Write a recommendation for the first person who asked and politely decline the second	☐	(0)
(-1)	☐	2.	Try to get to know them both a little better so you can make a more informed choice	☐	(+1)
(+1)	☐	3.	Follow your intuition about who deserves the recommendation more	☐	(-1)
(0)	☐	4.	Write them both recommendations anyway and let the selection committee decide	☐	(0)

2. Your client has asked for collaboration, but you suspect that she is not fully sharing her opinions and ideas. Your team needs buy-in from her on an initial set of recommendations to be able to move forward. What would you be most likely and least likely to do?

Least Likely				Most Likely	
(0)	☐	1.	Adopt a neutral presentation style to increase the odds your client will speak up	☐	(0)
(+1)	☐	2.	Present the recommendations confidently and trust that if you do your job, she will do hers	☐	(-1)
(-1)	☐	3.	State clearly upfront that you want to hear her reactions and ideas	☐	(+1)
(0)	☐	4.	Put in extra effort to make sure your team's recommendations are sound in case she remains reticent	☐	(0)

3. You are about to serve a new client and are getting advice from a peer who served them in the past. Among other things, your colleague shares that one of the women you will be

working closely with is highly competent, but he didn't like her tendency to frequently assert strong opinions. What would you be most likely and least likely to do?

Least Likely			Most Likely
(+1) ☐	1.	Be prepared to manage a potentially challenging client relationship	☐ (-1)
(0) ☐	2.	Take your colleague's feedback on board but wait before drawing any conclusions of your own	☐ (0)
(0) ☐	3.	Go out of your way early in the engagement to make sure you have earned her respect	☐ (0)
(-1) ☐	4.	Consider whether the same comment would be made about a male client and filter the feedback accordingly	☐ (+1)

4. You have just started serving a new client, and they have asked for your input to help assign client team members to your project. They have 8 qualified candidates and offered to provide you with whatever you need to formulate your input. What would you be most likely and least likely to request?

Least Likely			Most Likely
(-1) ☐	1.	Anonymized work history and a sense of strengths and growth areas for each candidate	☐ (+1)
(0) ☐	2.	Resumes and a brief written statement of each candidate's interest in the project	☐ (0)
(0) ☐	3.	A 15-minute individual interview with each of the candidates	☐ (0)
(+1) ☐	4.	Their judgment on who would be easiest to work with since all 8 are qualified	☐ (-1)

5. Your team is nearing the end of a long, intense [project] and deserves a team event to relax. You need someone to organize the activity. Eric and Liz are [junior colleagues] with similar workloads, but Liz has been with the team longer than Eric. Who would you be most likely and least likely to ask to plan the team event?

Least Likely			Most Likely
(0) ☐	1.	Eric, because he has been with the team the shortest	☐ (0)
(+1) ☐	2.	Liz, because she probably has better party-planning skills	☐ (-1)
(-1) ☐	3.	Suggest Eric, but let them both know it is okay to switch or share	☐ (+1)
(0) ☐	4.	Neither – volunteer yourself to do it instead	☐ (0)

6. Your team needs to scan and annotate a large number of your client's archived documents for a historical analysis. Carla said that she could do it, though you know she

has a lot of other work to do. Ben did not volunteer to help, but you don't think [he] is particularly busy right now. How would you be most likely and least likely to decide how to get this scanning done?

Least Likely			Most Likely
(0) ☐	1.	Ask a colleague on another team for an outside perspective on what they would do	☐ (0)
(0) ☐	2.	Assign the scanning to Ben so Carla can focus on her other work	☐ (0)
(+1) ☐	3.	Let Carla do it since she volunteered, as long as she can get her other work done	☐ (-1)
(-1) ☐	4.	Ask Carla and Ben for status reports on their other work before deciding	☐ (+1)

7. A friend is considering working with a former colleague who left the [organization] to join a start-up. You recall that colleague being highly knowledgeable and a strong problem-solver, but introverted and not well-liked by the team. What would you be most likely and least likely to advise?

Least Likely			Most Likely
(-1) ☐	1.	Factor into your advice whether the team would have liked the colleague better if not introverted	☐ (+1)
(0) ☐	2.	Share what you recall about your colleague as best as you can	☐ (0)
(+1) ☐	3.	Suggest avoiding working with the former colleague if your friend doesn't want a personality clash	☐ (-1)
(0) ☐	4.	Refrain from offering any advice since it is ultimately your friend's call	☐ (0)

8. Your team has put together a capability-building workshop for your client. You first want to train a select 15-20 employees who will become program ambassadors when you roll it out. You know most employees would enjoy being chosen. What process would you be most likely and least likely to use to select participants?

Least Likely			Most Likely
(0) ☐	1.	Ask your client to choose using whatever process makes the most sense to them	☐ (0)
(+1) ☐	2.	Ask managers to nominate a favorite member of their teams for this opportunity	☐ (-1)
(0) ☐	3.	Create a brief application process that can be shared with both employees and managers	☐ (0)
(-1) ☐	4.	Use anonymized applications and performance review data to select the best candidates	☐ (+1)

9. A teammate you have worked with for the past two months – though not that closely – has asked for your input into a 360-degree feedback survey for an upcoming learning program. Your sense is that your teammate is competent, but sometimes rubs people the wrong way. The feedback request form is anonymous, so you are free to share your honest opinions. What would you be most likely and least likely to do?

Least Likely			Most Likely
(0)	1.	Decline to participate, as you are just too busy with client work right now	(0)
(-1)	2.	Ask your teammate to send you some additional information so you can give better input	(+1)
(0)	3.	Share your limited observations as best and as objectively as you can	(0)
(+1)	4.	Offer specific, practical recommendations for how your teammate can improve the way they are perceived	(-1)

10. The executive director at a non-profit client you are working with is a former engineer who only recently moved into a leadership role. You are about to make your first presentation to the executive director. What would you be most likely and least likely to do?

Least Likely			Most Likely
(+1)	1.	Prepare as much as you can about the technical and operational details of your plan	(-1)
(0)	2.	Trust your experience and focus on making the best proposal you can	(0)
(0)	3.	Bring along client team members in case you get unexpected questions	(0)
(-1)	4.	Ask client team members for tips about what works well (or not) with the executive director	(+1)

Validation of Situational Judgment Test for Gender Inclusive Intentions

As part of this study, we created a new situational judgment test (SJT) to measure the extent to which individuals intend to behave in inclusive ways when responding to workplace situations where bias may arise.

Test Development. Situational judgment tests ask participants how they would respond to a range of context-specific scenarios as a way of detecting patterns in employees' motivations and behavioral tendencies across different circumstances (Lievens, Peeters, & Schollaert, 2008; Weekley & Ployhart, 2013). The SJT format was particularly well-suited for our study for several reasons: a) by presenting participants with multiple attractive options, SJTs can be more difficult to fake than traditional personality assessments (Hooper, Cullen, & Sackett, 2006); b) because the items are written in a way that is job-relevant, they also tend to have strong face validity and elicit positive reactions from respondents (Oostrom, De Soete, & Lievens, 2015); and c) SJT scores can be improved through organizationally-endorsed coaching without undermining their validity (Stemig, Sackett, & Lievens, 2015), which suggests they may capture a behavioral intention that is responsive to a training such as our intervention.

We developed our initial item pool by following a combination of critical-incident and theory-based methods, both of which have been shown to be effective methods for SJT development (Oostrom et al., 2015). To develop item stems that would be job-relevant for our population of employees, we solicited examples of common workplace situations where bias may arise from human resources and learning and development leaders at our partner organization. Our research team then turned these situations into an initial pool of 10 item stems and drafted theory-derived behavioral responses that ranged from bias-reinforcing to bias-reducing. These items were also improved thanks to input from our organizational partner. This

process resulted in a list of 10 questions asking employees what they would be most and least likely to do in different bias-prone situations from a list of four different response options ranging from bias-interrupting (earning a score of +1 if selected as most likely and a score of -1 if selected as least likely) to bias-reinforcing (earning a score of -1 if selected as most likely and a score of +1 if selected as least likely). Overall scores are determined by summing participants' scores across all 10 situations.

Validation Study. To demonstrate convergent validity with different measures of bias, as well as predictive validity for gender-inclusive behaviors, we collected data from a sample of working adults via Amazon Mechanical Turk, which typically provides samples more representative of the population than undergraduates (Buhrmester, Kwang, & Gosling, 2011). Participants were asked to complete our SJT and several scales commonly used in research this domain, and they agreed to be contacted for a follow-up survey one week later. The follow-up survey included the gender-career implicit association test (Greenwald, McGhee, & Schwartz, 1998) and multiple opportunities to engage in behaviors to promote gender inclusion, as described in more detail below.

Sample. A total of 299 participants completed the initial survey, and 243 (81.3%) of those respondents also completed the follow-up survey. The respondents in the final sample were 51% male and 81% Caucasian. The respondents had an average age of 35.6 years, and an average of 15 years of work experience. When asked to describe their political views, 58% described themselves as somewhat to extremely liberal, 19% were neither liberal nor conservative, and 23% said they were somewhat to extremely conservative.

Measures. Participants completed two rounds of surveys intended to measure the extent to which they hold biased attitudes and to what degree they were willing to engage in behaviors

that promote gender inclusion. The first survey included our SJT, along with modern sexism (Swim et al., 1995) and ambivalent sexism (Glick & Fiske, 1996), two commonly used measures of gender bias, as well as a measure of gender bias acknowledgment (described in detail under *Materials and Methods: Attitude Measures*). For exploratory purposes, we also included social dominance orientation (Pratto, Sidanius, Stallworth, & Malle, 1994), right-wing authoritarianism (Rattazzi, Bobbio, & Canova, 2007), a brief measure of Big 5 personality traits (Donnellan, Oswald, Baird, & Lucas, 2006), and a test of actively open-minded thinking (Baron, Scott, Fincher, & Metz, 2015).

At the one-week follow-up, participants completed the gender-career implicit association test and were offered an opportunity to advocate for women at a personal cost. Participants were told they would receive an additional $0.25 bonus above and beyond their payment for participating in this follow-up study. They were then told they could contribute some or all of this bonus to Ellevate, a professional development organization for women that aims to reduce gender inequality at work. Participants then indicated how much they would like to donate to Ellevate.

We also included several other measures that involved providing opinions or making public commitments to support gender equality.

- *Hiring Measure* – Participants were asked to evaluate a candidate for a managerial position in a task adapted from several prior studies (Bowles, Babcock, & Lai, 2007; Duguid & Thomas-Hunt, 2015; Rudman & Glick, 1999). Participants were randomly assigned to receive application materials from either a hypothetical male or a female candidate with equal qualifications. They then rated their likelihood of choosing that candidate, as well as the candidate's agentic and communal traits.

- *Discrimination Compensation* – In another task adapted from a prior study (Pietri et al., 2017), participants watched a brief video excerpt from a simulated job interview and learned that the job applicant had sued the prospective employer because she felt the interviewer asked several inappropriate questions about her pregnancy. Participants were asked how much the applicant should receive in damages if she wins her case. They could choose any amount between \$10,000 and \$100,000, which they were told was a typical award range in prior cases like this.
- *Advocating Measures* – Participants were given two opportunities to advocate for gender inclusiveness. First, participants were asked if they would be willing to sign the HeForShe Commitment, a United Nations Women's Solidarity Movement for Gender Equality. To increase the believability of this measure without asking for any identifiable information, we told them we would provide instructions for signing the pledge if they said "yes", asked them to write a short statement about the importance of the pledge, and asked if we could anonymously share their comment with future study participants. Second, participants were asked if they would be willing to share an article from *Harvard Business Review* about gender bias in entrepreneurship on social media if we provided them with a link to do so. We scored this measure by giving participants a 0 if they declined both invitations to advocate for gender inclusiveness, a 1 if they accepted one but not the other, and a 2 if they accepted both.

Results. To rule out nonresponse bias in our final sample (Rogelberg & Stanton, 2007), we compared participants who responded to both surveys with those who responded to only the first survey on each of the variables we measured in the first survey. Using independent samples t-tests, we found no significant differences between these groups on any of our bias-related

attitudinal measures (modern sexism, t(297) = 0.407, p = 0.685; ambivalent sexism, t(297) = 0.447, p = 0.655; gender bias acknowledgment, t(297) = 1.359, p = 0.175; racial bias acknowledgment, t(297) = 0.031, p = 0.976; authoritarian aggression, t(297) = -0.548, p = 0.585; conservatism, t(297) = -0.246, p = 0.806; social dominance orientation, t(297) = -0.015, p = 0.988) or on age (t(297) = -1.078, p = 0.282). Using chi-square tests, there were also no significant differences on gender ($X^2(2) = 0.743$, p = 0.690) or race ($X^2(4) = 7.203$, p = 0.126). Importantly, we also found no differences between groups on scores on our situational judgment test (t(297) = -0.416, p = 0.677). On the personality measures, participants who completed both surveys were significantly more conscientious (t(297) = -2.561, p = 0.012) and marginally less neurotic (t(297) = 1.936, p = 0.054) than those who completed only the first survey. There were no significant differences on agreeableness (t(297) = -0.595, p = 0.553), extraversion (t(297) = 0.975, p = 0.330), or openness (t(297) = 0.279, p = 0.780). Finally, the participants who completed both surveys scored significantly higher on actively open-minded thinking (t(297) = -2.526, p = 0.012) than those who completed only the first survey. These results suggest that our primary variables of interest (the bias measures) and demographics were unlikely to affect response rates, although a few of the exploratory personality measures we included might have.

We removed items nine and ten from our SJT due to heavily skewed responses. As expected, scores on our resulting 8-item SJT were significantly negatively correlated with both modern sexism (r = -0.189, p = 0.001) and ambivalent sexism (r = -0.235, p < 0.001), including the latter's sub-scales for both benevolent sexism (r = -0.187, p = 0.001) and hostile sexism (r = -0.177, p = 0.002). Scores on our SJT were also significantly negatively correlated with broader measures of biased attitudes, including social dominance orientation (r = -0.241, p < 0.001) and authoritarian aggression (r = -0.213, p < 0.001). Our SJT was significantly positively correlated

with gender bias acknowledgment ($r = 0.217$, $p < 0.001$) and racial bias acknowledgment ($r = 0.178$, $p = 0.002$), with the larger gap being driven by significant negative correlations between the SJT and self-ratings of gender bias ($r = -0.167$, $p = 0.004$) and racial bias ($r = -0.191$, $p = 0.001$). The SJT was not significantly associated with ratings of others' bias. A full set of correlations is reported in Table S25. Taken together, this suggests that higher scores on our SJT are associated with less biased attitudes.

To examine the unique role of our SJT in predicting future behavior, we conducted a hierarchical regression analysis with the amount of charitable contribution to Ellevate as our dependent variable. We entered the gender-related bias scales (benevolent sexism, hostile sexism, modern sexism, and gender bias acknowledgment) in the first step, the gender-career IAT in the second step, and our SJT in the third step. In the first two steps, none of the explicit or implicit biased attitude scales were significant predictors of the charitable contribution measure. In the third step, only the SJT was a significant predictor ($b = 0.003$, $p = 0.040$; see Table S26 for the full results of this analysis.) This suggests that although our SJT was correlated in the expected directions with other measures of biased attitudes, it was unique in its ability to predict this specific gender-inclusive behavior.

We ran similar regression analyses with each of the other behavioral measures and found mixed results. On the hiring measure, there were no significant differences in likelihood to work with a candidate based on the applicant's gender as determined by a one-way ANOVA ($F(1, 243) = 1.617$, $p = 0.205$). Of our attitude measures, the only significant predictors of likelihood to work with a candidate were benevolent sexism ($b = 0.246$, $p = 0.017$), actively open-minded thinking ($b = -0.302$, $p = 0.007$) and neuroticism ($b = -.132$, $p = .050$), all of which remained significant when controlling for the gender of the applicant but went in the opposite of the

hypothesized direction. On the discrimination measure, modern sexism (b = -4069.91, p = 0.050) and gender bias acknowledgment (b = 2837.15, p = 0.034) were significant predictors of the sum of money participants would award to the woman in the video who was suing a prospective employer for discrimination. For the advocating measure, results of an ordinal regression analysis showed modern sexism (b = -0.541, p = .002) to be the only significant predictor of participants' willingness to sign a petition and/or share an article on social media.

We also tested whether our SJT predicted our charitable contribution measure above and beyond the personality traits we collected by entering them into a hierarchical regression analysis with actively open-minded thinking in the first step, each of the Big 5 personality traits in the second step, and our SJT in the third step. In the first step, actively open-minded thinking was not a significant predictor of the financial contribution to a woman's cause (b = -0.009, p = 0.181). In the second step, actively open-minded thinking (b = -0.016, p = 0.027) and agreeableness (b = 0.014, p = 0.007) were significant predictors of the financial contribution, although actively open-minded thinking was in the opposite of the expected direction. In the third step, actively open-minded thinking (b = -0.020, p = 0.007) and agreeableness (b = 0.012, p = 0.015) remained significant, but the SJT was also a significant predictor of charitable contribution (b = 0.004, p = 0.028; see Table S27 for the full results of this analysis). The other Big 5 personality traits were insignificant. This suggests that although our SJT is positively associated with agreeableness and AOT, it still has incremental predictive value for future gender-inclusive behaviors like our charitable contribution measure.

Lastly, to test whether our SJT still held incremental predictive value after controlling for both biased attitudes and personality measures, we conducted a hierarchical regression analysis with the gender-related bias scales in the first step, the gender-career IAT in the second step, the

Big 5 personality dimensions and actively open-minded thinking in the third step, and our SJT in the fourth step. In the first two steps, neither the gender-related bias scales nor the IAT were significant predictors of financial contribution to a women's cause. In the third step, agreeableness ($\beta = 0.012$, $p = 0.032$) was a significant predictor and actively open-minded thinking was a marginally negative predictor ($\beta = -0.016$, $p = 0.052$). In the final step, the SJT significantly predicted financial contribution ($\beta = 0.004$, $p = 0.037$), with agreeableness ($\beta = 0.011$, $p = 0.049$) and actively open-minded thinking ($\beta = -0.018$, $p = 0.023$; see Table S28 for the full results of this analysis) remaining significant.

FIGURES

FIG. S1

Study Timeline

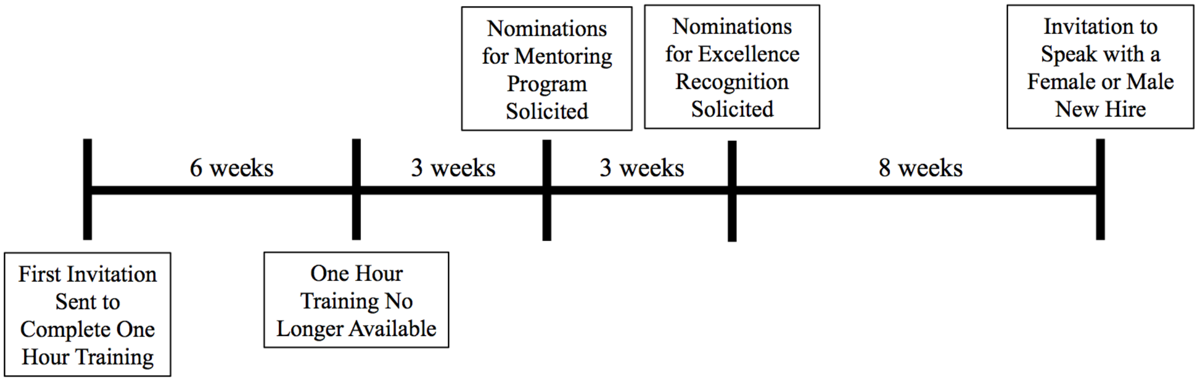

FIG. S2

Relative Levels of Attitudinal Support for Women by Demographic Subgroup in the Control Condition

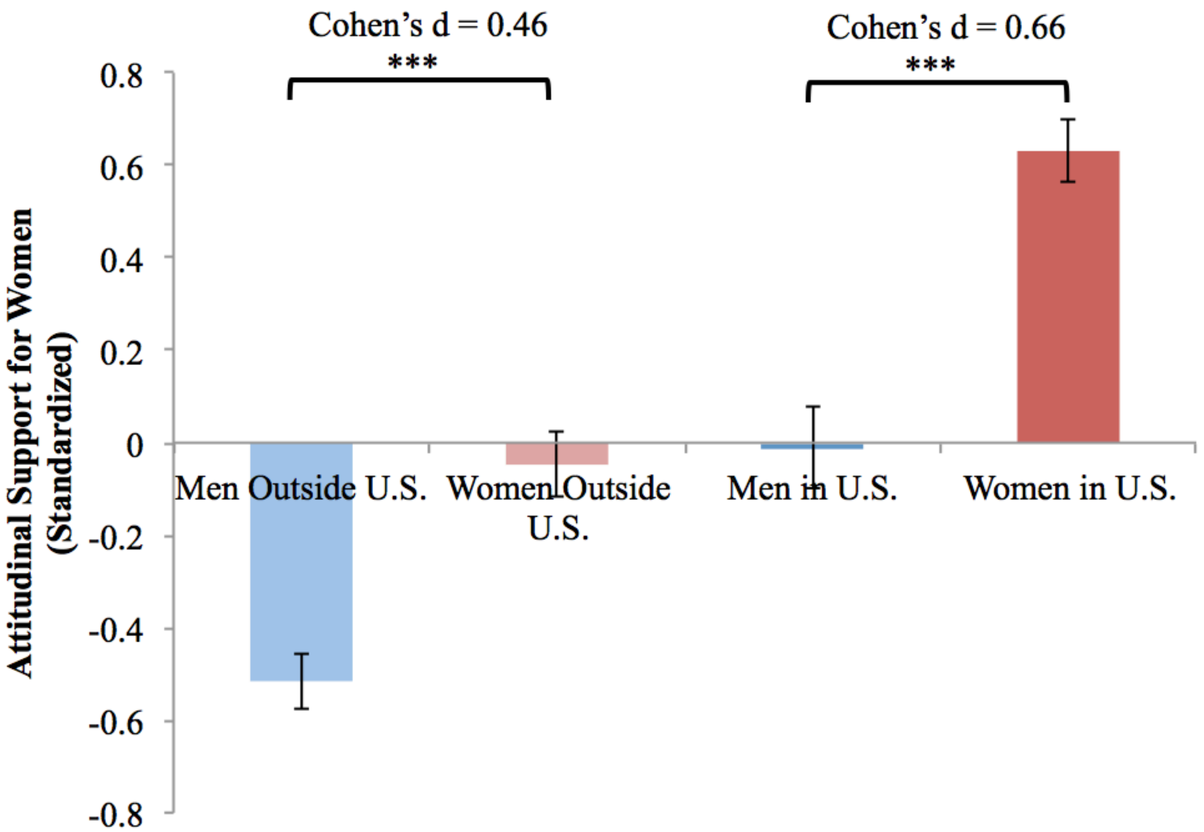

Note. Due to confidentiality requirements of our field partner, we do not report absolute unstandardized levels of this measure. Brackets and significance levels report differences between specified groups.
*, **, *** and denote significance at the 5%, 1%, and 0.1% levels, respectively

FIG. S3

Scatterplots (with Trend Lines Weighted by Country and Gender Subgroup Size) Depicting the Raw Relationship Between Subgroups' Attitudinal Support for Women in the Absence of Training and Treatment's Impact on Attitudes and Behaviors

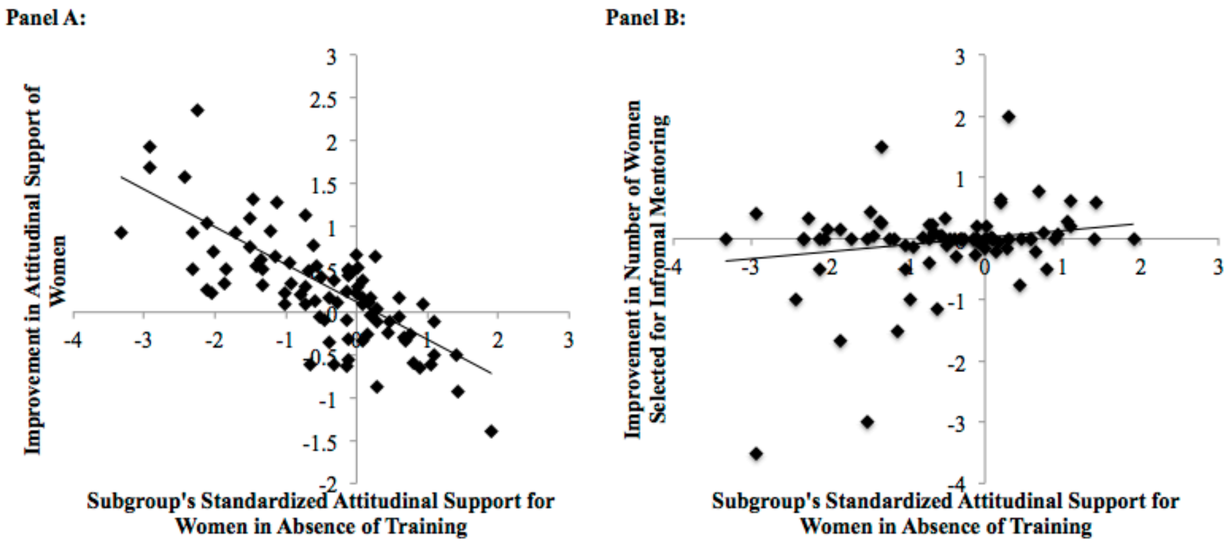

Note. These figures depict scatterplots of the relationship between our proxy for pre-training attitudes and the intervention's impact on attitudes and behaviors. Each point represents a country-gender demographic subgroup (e.g., women in France). Our proxy for pre-training attitudes is the demographic subgroup's average level of attitudinal support for women in the control condition. Trend lines have been weighted by subgroup size.

TABLES

TABLE S1

Summary of Differences Between Gender-Bias and General-Bias Trainings on Attitudes Pertaining to Women

	Difference in Attitude Measures (Gender-Bias – General-Bias)		
**Demographic Subgroup**	**Attitudinal Support for Women** *(Higher Scores Reflect More Supportive Attitudes)*	**Gender Bias Acknowledgment** *(Higher Scores Reflect Smaller Gap)*	**Gender Inclusive Intentions** *(Higher Scores Reflect Higher Intentions to Be Inclusive)*
**Overall**	0.0162<br>(0.0484)<br>N = 1,544	-0.0357<br>(0.0493)<br>N = 1,548	0.0495<br>(0.0534)<br>N = 1,523
Men	-0.0150<br>(0.0627)<br>N = 938	-0.0663<br>(0.0631)<br>N = 941	0.0196<br>(0.0680)<br>N = 923
Women	0.0907<br>(0.0698)<br>N = 606	-0.000996<br>(0.0781)<br>N = 606	0.104<br>(0.858)<br>N = 600
Employees Outside the U.S.	-0.00171<br>(0.0626)<br>N = 914	-0.0963<br>(0.0660)<br>N = 915	0.0490<br>(0.0684)<br>N = 898
Employees in the U.S.	0.0210<br>(0.0730)<br>N = 630	0.0569<br>(0.0737)<br>N = 633	0.0432<br>(0.0853)<br>N = 625
Men Outside the U.S.	-0.0819<br>(0.0787)<br>N = 574	-0.131<br>(0.0830)<br>N = 575	0.0342<br>(0.0871)<br>N = 562
Women Outside the U.S.	0.127<br>(0.0954)<br>N = 340	-0.0344<br>(0.108)<br>N = 340	0.0702<br>(0.110)<br>N = 336
Men in the U.S.	0.0449<br>(0.100)<br>N = 364	0.0446<br>(0.0970)<br>N = 366	-0.0169<br>(0.109)<br>N = 361
Women in the U.S.	0.0571<br>(0.0978)<br>N = 266	0.0381<br>(0.113)<br>N = 267	0.0152<br>(0.136)<br>N = 264

Note. This table reports differences between the gender-bias and general-bias trainings, standard errors for these differences in parentheses, and sample sizes for the specified measures split by demographic subgroup. Statistical significance reported is from t-tests. †, *, **, and *** denote significance at the 10%, 5%, 1%, and 0.1% levels, respectively

TABLE S2

Summary of Differences Between Gender-Bias and General-Bias Trainings on Behaviors Pertaining to Women

	**Difference in Behavior Measures (Gender-Bias – General-Bias)**		
**Demographic Subgroup**	**Number of Women Selected for Informal Mentoring**	**Number of Women Nominated for Excellence Award**	**Difference in % Volunteering to Talk to a Female vs. Male New Hire**
**Overall**	-0.00691<br>(0.0296)<br>N = 2,003	0.00630<br>(0.00570)<br>N = 2,003	0.0558<br>(0.0442)<br>N = 1,932
Men	-0.00103<br>(0.0300)<br>N = 1,230	-0.00282<br>(0.00668)<br>N = 1,230	0.0266<br>(0.0567)<br>N = 1,197
Women	-0.00689<br>(0.0599)<br>N = 773	0.0215*<br>(0.0102)<br>N = 773	0.113<br>(0.0706)<br>N = 735
Employees Outside the U.S.	-0.0245<br>(0.0362)<br>N = 1,221	0.00449<br>(0.00631)<br>N = 1,221	0.0626<br>(0.0571)<br>N = 1,166
Employees in the U.S.	0.0200<br>(0.0506)<br>N = 782	0.00898<br>(0.0108)<br>N = 782	0.0466<br>(0.0700)<br>N = 766
Men Outside the U.S.	-0.0338<br>(0.0373)<br>N = 775	-0.00303<br>(0.00576)<br>N = 775	0.0880<br>(0.0722)<br>N = 750
Women Outside the U.S.	-0.00390<br>(0.0750)<br>N = 446	0.0185<br>(0.0140)<br>N = 446	0.0376<br>(0.0932)<br>N = 416
Men in the U.S.	0.0553<br>(0.0505)<br>N = 455	-0.00320<br>(0.0152)<br>N = 455	-0.0676<br>(0.0918)<br>N = 447
Women in the U.S.	-0.00966<br>(0.0979)<br>N = 327	0.0255†<br>(0.0148)<br>N = 327	0.221*<br>(0.108)<br>N = 319

Note. This table reports differences between the gender-bias and general-bias trainings, standard errors for these differences in parentheses, and sample sizes for the specified measures split by demographic subgroup. Statistical significance reported is from t-tests for the informal mentoring program and recognition for excellence program. Statistical significance is reported from a regression with no controls for the audit study.
†, *, **, and *** denote significance at the 10%, 5%, 1%, and 0.1% levels, respectively

TABLE S3

Summary of Differences Between Gender-Bias and General-Bias Trainings on Attitudes and Behaviors Pertaining to Racial Minorities

	**Difference in Measures (Gender-Bias – General-Bias)**		
**Demographic Subgroup**	**Racial Bias Acknowledgment** *(Higher Scores Reflect Smaller Gap)*	**Number of Racial Minorities Selected for Informal Mentoring**	**Number of Racial Minorities Nominated for Excellence Award**
**Overall**	-0.100*<br>(0.0492)<br>N = 1,548	-<br>-<br>-	-<br>-<br>-
**Employees in the U.S.**	-0.0386<br>(0.0760)<br>N = 633	0.0232<br>(0.0405)<br>N = 782	0.0189<br>(0.0119)<br>N = 782
Men in the U.S.	-0.0628<br>(0.105)<br>N = 366	0.0323<br>(0.0528)<br>N = 455	0.0173<br>(0.0179)<br>N = 455
Women in the U.S.	0.00116<br>(0.110)<br>N = 267	0.0137<br>(0.0634)<br>N = 327	0.0192<br>(0.0136)<br>N = 327
Whites in the U.S.	-0.0509<br>(0.0954)<br>N = 388	0.0166<br>(0.0372)<br>N = 461	0.0158†<br>(0.00867)<br>N = 461
Racial Minorities in the U.S.	-0.00544<br>(0.126)<br>N = 245	0.0571<br>(0.0827)<br>N = 321	0.0316<br>(0.0259)<br>N = 321

Note. This table reports differences between the gender-bias and general-bias trainings, standard errors for these differences in parentheses, and sample sizes for the specified measures split by demographic subgroup. Statistical significance reported is from t-tests. †, *, **, and *** denote significance at the 10%, 5%, 1%, and 0.1% levels, respectively

TABLE S4

Summary of Differences Between Gender-Bias and Control Trainings on Attitudes Pertaining to Women

	Difference in Attitude Measures (Gender-Bias – Control)		
**Demographic Subgroup**	**Attitudinal Support for Women** *(Higher Scores Reflect More Supportive Attitudes)*	**Gender Bias Acknowledgment** *(Higher Scores Reflect Smaller Gap)*	**Gender Inclusive Intentions** *(Higher Scores Reflect Higher Intentions to Be Inclusive)*
**Overall**	0.171***<br>(0.0519)<br>N = 1,574	0.187***<br>(0.0513)<br>N = 1,577	0.151**<br>(0.0500)<br>N = 1,542
Men	0.208**<br>(0.0672)<br>N = 970	0.143*<br>(0.0626)<br>N = 972	0.138*<br>(0.0635)<br>N = 949
Women	0.118<br>(0.0731)<br>N = 604	0.254**<br>(0.0869)<br>N = 605	0.171*<br>(0.0800)<br>N = 593
Employees Outside the U.S.	0.266***<br>(0.0656)<br>N = 939	0.150*<br>(0.0673)<br>N = 939	0.197**<br>(0.0643)<br>N = 914
Employees in the U.S.	-0.00937<br>(0.0785)<br>N = 635	0.246**<br>(0.0793)<br>N = 638	0.0698<br>(0.0787)<br>N = 628
Men Outside the U.S.	0.210*<br>(0.0834)<br>N = 606	0.108<br>(0.0807)<br>N = 606	0.175*<br>(0.0799)<br>N = 588
Women Outside the U.S.	0.308**<br>(0.0960)<br>N = 333	0.252*<br>(0.119)<br>N = 333	0.218*<br>(0.108)<br>N = 326
Men in the U.S.	0.111<br>(0.108)<br>N = 364	0.207*<br>(0.100)<br>N = 366	0.0523<br>(0.105)<br>N = 361
Women in the U.S.	-0.0757<br>(0.103)<br>N = 271	0.253*<br>(0.127)<br>N = 272	0.130<br>(0.118)<br>N = 267

Note. This table reports differences between the gender-bias and control trainings, standard errors for these differences in parentheses, and sample sizes for the specified measures split by demographic subgroup. Statistical significance reported is from t-tests. †, *, **, and *** denote significance at the 10%, 5%, 1%, and 0.1% levels, respectively

TABLE S5

Summary of Differences Between Gender-Bias and Control Trainings on Behaviors Pertaining to Women

	Difference in Behavior Measures (Gender-Bias – Control)		
**Demographic Subgroup**	**Number of Women Selected for Informal Mentoring**	**Number of Women Nominated for Excellence Award**	**Difference in % Volunteering to Talk to a Female vs. Male New Hire**
**Overall**	0.00357<br>(0.0285)<br>N = 2,032	0.00665<br>(0.00544)<br>N = 2,032	0.0621<br>(0.0439)<br>N = 1,947
Men	-0.0137<br>(0.0315)<br>N = 1,267	0.00603<br>(0.00497)<br>N = 1,267	0.00778<br>(0.0557)<br>N = 1,233
Women	0.0351<br>(0.0547)<br>N = 765	0.00835<br>(0.0118)<br>N = 765	0.165*<br>(0.0710)<br>N = 714
Employees Outside the U.S.	-0.0582<br>(0.0379)<br>N = 1,252	0.000230<br>(0.00673)<br>N = 1,252	0.0653<br>(0.0560)<br>N = 1,192
Employees in the U.S.	0.104*<br>(0.0425)<br>N = 780	0.0169†<br>(0.00916)<br>N = 780	0.0571<br>(0.0709)<br>N = 755
Men Outside the U.S.	-0.0443<br>(0.0400)<br>N = 825	0.000271<br>(0.00484)<br>N = 825	0.0581<br>(0.0693)<br>N = 802
Women Outside the U.S.	-0.0938<br>(0.0796)<br>N = 427	-0.00200<br>(0.0173)<br>N = 427	0.106<br>(0.0947)<br>N = 390
Men in the U.S.	0.0453<br>(0.0515)<br>N = 442	0.0154<br>(0.0111)<br>N = 442	-0.0632<br>(0.0950)<br>N = 431
Women in the U.S.	0.192**<br>(0.0716)<br>N = 338	0.0203<br>(0.0155)<br>N = 338	0.252*<br>(0.107)<br>N = 324

Note. This table reports differences between the gender-bias and control trainings, standard errors for these differences in parentheses, and sample sizes for the specified measures split by demographic subgroup. Statistical significance reported is from t-tests for the informal mentoring program and recognition for excellence program. Statistical significance is reported from a regression with no controls for the audit study.
†, *, **, and *** denote significance at the 10%, 5%, 1%, and 0.1% levels, respectively

TABLE S6

Summary of Differences Between Gender-Bias and Control Trainings on Measures Pertaining to Racial Minorities

	**Difference in Measures (Gender-Bias – Control)**		
**Demographic Subgroup**	**Racial Bias Acknowledgment** *(Higher Scores Reflect Smaller Gap)*	**Number of Racial Minorities Selected for Informal Mentoring**	**Number of Racial Minorities Nominated for Excellence Award**
**Overall**	0.138**<br>(0.0517)<br>N = 1,577	-<br>-<br>-	-<br>-<br>-
**Employees in the U.S.**	0.174*<br>(0.0798)<br>N = 638	0.0624†<br>(0.0367)<br>N = 780	0.0268*<br>(0.0104)<br>N = 780
Men in the U.S.	0.212†<br>(0.109)<br>N = 366	0.0361<br>(0.0511)<br>N = 442	0.0309*<br>(0.0155)<br>N = 442
Women in the U.S.	0.143<br>(0.117)<br>N = 272	0.0944†<br>(0.0526)<br>N = 338	0.0196<br>(0.0132)<br>N = 338
Whites in the U.S.	0.0919<br>(0.0970)<br>N = 407	0.0526†<br>(0.0302)<br>N = 480	0.00700<br>(0.0102)<br>N = 480
Racial Minorities in the U.S.	0.317*<br>(0.139)<br>N = 231	0.0867<br>(0.0815)<br>N = 300	0.0600**<br>(0.0215)<br>N = 300

Note. This table reports differences between the gender-bias and control trainings, standard errors for these differences in parentheses, and sample sizes for the specified measures split by demographic subgroup. Statistical significance reported is from t-tests.

†, *, **, and *** denote significance at the 10%, 5%, 1%, and 0.1% levels, respectively

TABLE S7

Summary of Differences Between General-Bias and Control Trainings on Attitudes Pertaining to Women

	**Difference in Attitude Measures General-Bias – Control)**		
**Demographic Subgroup**	**Attitudinal Support for Women** *(Higher Scores Reflect More Supportive Attitudes)*	**Gender Bias Acknowledgment** *(Higher Scores Reflect Smaller Gap)*	**Gender Inclusive Intentions** *(Higher Scores Reflect Higher Intentions to Be Inclusive)*
**Overall**	0.155** (0.0516) N = 1,536	0.223*** (0.0508) N = 1,539	0.101* (0.0501) N = 1,499
Men	0.223** (0.0681) N = 930	0.209*** (0.0629) N = 931	0.118† (0.0644) N = 908
Women	0.0277 (0.0713) N = 606	0.255** (0.0837) N = 608	0.0665 (0.0795) N = 591
Employees Outside the U.S.	0.267*** (0.0649) N = 935	0.246*** (0.0664) N = 936	0.148* (0.0633) N = 908
Employees in the U.S.	-0.0303 (0.0781) N = 601	0.189* (0.0785) N = 603	0.0266 (0.0811) N = 591
Men Outside the U.S.	0.292*** (0.0824) N = 606	0.239** (0.0796) N = 607	0.140 (0.0787) N = 588
Women Outside the U.S.	0.182† (0.0984) N = 329	0.286* (0.117) N = 329	0.148 (0.106) N = 320
Men in the U.S.	0.0663 (0.115) N = 324	0.162 (0.102) N = 324	0.0691 (0.111) N = 320
Women in the U.S.	-0.133 (0.0918) N = 277	0.215† (0.120) N = 279	-0.0219 (0.119) N = 271

Note. This table reports differences between the general-bias and control trainings, standard errors for these differences in parentheses, and sample sizes for the specified measures split by demographic subgroup. Statistical significance reported is from t-tests. †, *, **, and *** denote significance at the 10%, 5%, 1%, and 0.1% levels, respectively

TABLE S8

Summary of Differences Between General-Bias and Control Trainings on Behaviors Pertaining to Women

	Difference in Behavior Measures General-Bias – Control)		
**Demographic Subgroup**	**Number of Women Selected for Informal Mentoring**	**Number of Women Nominated for Excellence Award**	**Difference in % Volunteering to Talk to a Female vs. Male New Hire**
**Overall**	0.0105<br>(0.0295)<br>N = 1,987	0.000353<br>(0.00511)<br>N = 1,987	0.00628<br>(0.0444)<br>N = 1,917
Men	-0.0127<br>(0.0314)<br>N = 1,207	0.00885<br>(0.00595)<br>N = 1,207	-0.0188<br>(0.0570)<br>N = 1,180
Women	0.0420<br>(0.0570)<br>N = 780	-0.0132<br>(0.00917)<br>N = 780	0.0519<br>(0.0707)<br>N = 737
Employees Outside the U.S.	-0.0337<br>(0.0392)<br>N = 1,231	-0.00426<br>(0.00626)<br>N = 1,231	0.00266<br>(0.0566)<br>N = 1,176
Employees in the U.S.	0.0840†<br>(0.0436)<br>N = 756	0.00787<br>(0.00874)<br>N = 756	0.0105<br>(0.0716)<br>N = 741
Men Outside the U.S.	-0.0105<br>(0.0418)<br>N = 798	0.00330<br>(0.00560)<br>N = 798	-0.0298<br>(0.0707)<br>N = 776
Women Outside the U.S.	-0.0899<br>(0.0806)<br>N = 433	-0.0205<br>(0.0145)<br>N = 433	0.0685<br>(0.0946)<br>N = 400
Men in the U.S.	-0.0100<br>(0.0445)<br>N = 409	0.0186<br>(0.0138)<br>N = 409	0.00441<br>(0.0975)<br>N = 404
Women in the U.S.	0.201*<br>(0.0788)<br>N = 347	-0.00522<br>(0.00997)<br>N = 347	0.0314<br>(0.107)<br>N = 337

Note. This table reports differences between the general-bias and control trainings, standard errors for these differences in parentheses, and sample sizes for the specified measures split by demographic subgroup. Statistical significance reported is from t-tests for the informal mentoring program and recognition for excellence program. Statistical significance is reported from a regression with no controls for the audit study.

†, *, **, and *** denote significance at the 10%, 5%, 1%, and 0.1% levels, respectively

TABLE S9

Summary of Differences Between General-Bias and Control Trainings on Attitudes and Behaviors Pertaining to Racial Minorities

	**Difference in Measures (General-Bias – Control)**		
**Demographic Subgroup**	**Racial Bias Acknowledgment** *(Higher Scores Reflect Smaller Gap)*	**Number of Racial Minorities Selected for Informal Mentoring**	**Number of Racial Minorities Nominated for Excellence Award**
**Overall**	0.238<br>(0.0505)<br>N = 1,539	-<br>-<br>-	-<br>-<br>-
**Employees in the U.S.**	0.212**<br>(0.0760)<br>N = 603	0.0392<br>(0.0344)<br>N = 756	0.00787<br>(0.00874)<br>N = 756
Men in the U.S.	0.275*<br>(0.107)<br>N = 324	0.00388<br>(0.0496)<br>N = 409	0.0136<br>(0.0146)<br>N = 409
Women in the U.S.	0.142<br>(0.108)<br>N = 279	0.0807†<br>(0.0469)<br>N = 347	0.000366<br>(0.00816)<br>N = 347
Whites in the U.S.	0.143<br>(0.0945)<br>N = 363	0.0361<br>(0.0292)<br>N = 435	-0.00881<br>(0.00649)<br>N = 435
Racial Minorities in the U.S.	0.322*<br>(0.127)<br>N = 240	0.0296<br>(0.0703)<br>N = 321	0.0284<br>(0.0186)<br>N = 321

Note. This table reports differences between the general-bias and control trainings, standard errors for these differences in parentheses, and sample sizes for the specified measures split by demographic subgroup. Statistical significance reported is from t-tests.
†, *, **, and *** denote significance at the 10%, 5%, 1%, and 0.1% levels, respectively

TABLE S10

Balance Checks Suggest That Randomization to Experimental Conditions Was Successful

	**Control Group (N = 1,010)**	**Treatment Group (N = 2,006)**	**Significance**
Percent Male	61.7%	61.4%	p = 0.874 (proportions test)
Percent in U.S.	37.4%	39.0%	p = 0.393 (proportions test)
Percent Male Outside of U.S.	42.0%	38.6%	p = 0.0764 (proportions test)
Percent Female Outside of U.S.	20.5%	22.2%	p = 0.275 (proportions test)
Percent Male in U.S.	19.6%	22.7%	p = 0.0527 (proportions test)
Percent Female in U.S.	17.7%	16.3%	p = 0.324 (proportions test)
Overall Completion Rate	75.1%	75.9%	p = 0.640 (proportions test)
Completion Rate of Men Outside of U.S.	72.4%	72.5%	p = 0.967 (proportions test)
Completion Rate of Women Outside of U.S.	74.9%	75.3%	p = 0.900 (proportions test)
Completion Rate of Men in U.S.	80.8%	79.3%	p = 0.668 (proportions test)
Completion Rate of Women in U.S.	76.5%	80.7%	p = 0.266 (proportions test)
Average Rating of Training on a Scale from 1 (Not at all valuable) to 7 (Very valuable)	5.27 (SD = 1.33)	4.93 (SD = 1.39)	p < 0.0001 (t-test)
Median Completion Time	70.7 minutes	65.5 minutes	p = 0.016 (Mood's median test)

TABLE S11

Summary of Differences Between Treatment and Control Conditions on Attitudes Pertaining to Women

	Difference in Attitude Measures (Treatment – Control)		
**Demographic Subgroup**	**Attitudinal Support for Women** *(Higher Scores Reflect More Supportive Attitudes)*	**Gender Bias Acknowledgment** *(Higher Scores Reflect Smaller Gap)*	**Gender Inclusive Intentions** *(Higher Scores Reflect Higher Intentions to Be Inclusive)*
**Overall**	0.163***<br>(0.0438)<br>N = 2,327	0.204***<br>(0.0436)<br>N = 2,332	0.126**<br>(0.0444)<br>N = 2,282
Men	0.215***<br>(0.0569)<br>N = 1,419	0.175**<br>(0.0543)<br>N = 1,422	0.129*<br>(0.0564)<br>N = 1,390
Women	0.0729<br>(0.0619)<br>N = 908	0.255***<br>(0.0718)<br>N = 910	0.119†<br>(0.0712)<br>N = 892
Employees Outside the U.S.	0.266***<br>(0.0553)<br>N = 1,394	0.198***<br>(0.0572)<br>N = 1,395	0.172**<br>(0.0563)<br>N = 1,360
Employees in the U.S.	-0.0193<br>(0.0666)<br>N = 933	0.219**<br>(0.0673)<br>N = 937	0.0495<br>(0.0714)<br>N = 922
Men Outside the U.S.	0.251***<br>(0.0695)<br>N = 893	0.173*<br>(0.0691)<br>N = 894	0.158*<br>(0.0699)<br>N = 869
Women Outside the U.S.	0.246**<br>(0.0845)<br>N = 501	0.269**<br>(0.100)<br>N = 501	0.183†<br>(0.0949)<br>N = 491
Men in the U.S.	0.0912<br>(0.0944)<br>N = 526	0.187*<br>(0.0879)<br>N = 528	0.0597<br>(0.0953)<br>N = 521
Women in the U.S.	-0.105<br>(0.0835)<br>N = 407	0.234*<br>(0.103)<br>N = 409	0.0528<br>(0.107)<br>N = 401

Note. This table reports differences between the treatment and control conditions, standard errors for these differences in parentheses, and sample sizes for the specified measures split by demographic subgroup. Statistical significance reported is from t-tests.
†, *, **, and *** denote significance at the 10%, 5%, 1%, and 0.1% levels, respectively

TABLE S12

Summary of Differences Between Treatment and Control Conditions on Behaviors Pertaining to Women

	**Difference in Behavior Measures (Treatment – Control)**		
**Demographic Subgroup**	**Number of Women Selected for Informal Mentoring**	**Number of Women Nominated for Excellence Award**	**Difference in % Volunteering to Talk to a Female vs. Male New Hire**
**Overall**	0.00700<br>(0.0252)<br>N = 3,016	0.00357<br>(0.00468)<br>N = 3,016	0.0346<br>(0.0382)<br>N = 2,898[a]
Men	-0.0132<br>(0.0268)<br>N = 1,852	0.00737<br>(0.00509)<br>N = 1,852	-0.00491<br>(0.0487)<br>N = 1,805
Women	0.0386<br>(0.0496)<br>N = 1,159	-0.00262<br>(0.00906)<br>N = 1,159	0.106†<br>(0.0614)<br>N = 1,093
Employees Outside the U.S.	-0.0461<br>(0.0326)<br>N = 1,852	-0.00198<br>(0.00555)<br>N = 1,852	0.0345<br>(0.0485)<br>N = 1,767
Employees in the U.S.	0.0943*<br>(0.0399)<br>N = 1,159	0.0125<br>(0.00838)<br>N = 1,159	0.0344<br>(0.0620)<br>N = 1,131
Men Outside the U.S.	-0.0280<br>(0.0339)<br>N = 1,199	0.00173<br>(0.00461)<br>N = 1,199	0.0155<br>(0.0599)<br>N = 1,164
Women Outside the U.S.	-0.0919<br>(0.0687)<br>N = 653	-0.0114<br>(0.0134)<br>N = 653	0.0851<br>(0.0822)<br>N = 603
Men in the U.S.	0.0196<br>(0.0436)<br>N = 653	0.0169<br>(0.0119)<br>N = 653	-0.0314<br>(0.0844)<br>N = 641
Women in the U.S.	0.196**<br>(0.0708)<br>N = 506	0.00718<br>(0.0116)<br>N = 506	0.139<br>(0.0927)<br>N = 490

Note. This table reports differences between the treatment and control, standard errors for these differences in parentheses, and sample sizes for the specified measures split by demographic subgroup. Statistical significance reported is from t-tests for the informal mentoring program and recognition for excellence program. Statistical significance is reported from a regression with no controls for the audit study. We do not have the gender or office location of five participants, so we include them in the overall intention-to-treat

analysis, but we cannot include them in demographic subgroup analyses.
[a]We only include participants who received an email about talking to a new hire in this intention-to-treat analysis. Some participants in our study were not on the original invite list (e.g., due to people forwarding the training to others), so they did not receive any of the emails for follow-up behavioral measures. For the informal mentoring program and the recognition for excellence program, we can treat these people as nominating zero women; however, because the dependent variable for the audit study is the difference in the willingness to talk to the female versus male new hire, if a participant did not receive any email, they were not randomly assigned to speak to either a female or a male new hire, so we cannot count them in either bucket. We still conduct an intention-to-treat analysis for all participants who received any email about speaking to a new hire.
†, *, **, and *** denote significance at the 10%, 5%, 1%, and 0.1% levels, respectively

TABLE S13

Estimated Treatment Effects on Attitudes Pertaining to Women Using Standard OLS Regression Specifications for Different Subgroups

	**Estimated Treatment Effect**		
**Demographic Subgroup**	**Attitudinal Support for Women** *(Higher Scores Reflect More Supportive Attitudes)*	**Gender Bias Acknowledgment** *(Higher Scores Reflect Smaller Gap)*	**Gender Inclusive Intentions** *(Higher Scores Reflect Higher Intentions to Be Inclusive)*
**Overall**	0.143*** (0.0413) N = 2,327	0.211*** (0.0440) N = 2,332	0.146** (0.0454) N = 2,282
Men	0.191*** (0.0563) N = 1,419	0.179** (0.0557) N = 1,422	0.156** (0.0582) N = 1,390
Women	0.00598 (0.0621) N = 908	0.314*** (0.0759) N = 910	0.136† (0.0760) N = 892
Employees Outside the U.S.	0.239*** (0.0535) N = 1,394	0.218*** (0.0578) N = 1,395	0.203*** (0.0582) N = 1,360
Employees in the U.S.	0.00813 (0.0659) N = 933	0.203** (0.0680) N = 937	0.0543 (0.0734) N = 922
Men Outside the U.S.	0.245*** (0.0695) N = 893	0.182* (0.0712) N = 894	0.208** (0.0729) N = 869
Women Outside the U.S.	0.131 (0.0895) N = 501	0.396*** (0.106) N = 501	0.231* (0.103) N = 491
Men in the U.S.	0.0953 (0.0984) N = 526	0.181* (0.0904) N = 528	0.0687 (0.0989) N = 521
Women in the U.S.	-0.107 (0.0864) N = 407	0.214* (0.108) N = 409	0.0136 (0.114) N = 401

Note. This table reports estimated treatment effects and standard errors (in parentheses) from ordinary least squares regressions predicting the specified dependent variable using fixed effects for office location, job category, race, and gender. Each regression includes only participants from the demographic subgroup specified.
†, *, **, and *** denote significance at the 10%, 5%, 1%, and 0.1% levels, respectively

TABLE S14

Estimated Treatment Effects on Behaviors Pertaining to Women Using Standard OLS Regression Specifications for Different Subgroups

	**Estimated Treatment Effect**		
**Demographic Subgroup**	**Number of Women Selected for Informal Mentoring**	**Number of Women Nominated for Excellence Award**	**Difference in % Volunteering to Talk to a Female vs. Male New Hire**
**Overall**	0.00855<br>(0.0277)<br>N = 3,011	0.00273<br>(0.00531)<br>N = 3,011	0.0419<br>(0.0388)<br>N = 2,898[a]
Men	-0.0221<br>(0.0277)<br>N = 1,852	0.00793<br>(0.00511)<br>N = 1,852	0.00977<br>(0.0499)<br>N = 1,805
Women	0.0480<br>(0.0589)<br>N = 1,159	-0.00224<br>(0.0116)<br>N = 1,159	0.134*<br>(0.0665)<br>N = 1,093
Employees Outside the U.S.	-0.0539<br>(0.0372)<br>N = 1,852	-0.00452<br>(0.00704)<br>N = 1,852	0.0494<br>(0.0496)<br>N = 1,767
Employees in the U.S.	0.103*<br>(0.0392)<br>N = 1,159	0.0124†<br>(0.00655)<br>N = 1,159	0.0353<br>(0.0633)<br>N = 1,131
Men Outside the U.S.	-0.0399<br>(0.0407)<br>N = 1,199	0.00334<br>(0.00504)<br>N = 1,199	0.0259<br>(0.0618)<br>N = 1,164
Women Outside the U.S.	-0.101<br>(0.0837)<br>N = 653	-0.00939<br>(0.0154)<br>N = 653	0.124<br>(0.0922)<br>N = 603
Men in the U.S.	0.00701<br>(0.0247)<br>N = 653	0.0153<br>(0.0121)<br>N = 653	0.00873<br>(0.0871)<br>N = 641
Women in the U.S.	0.212**<br>(0.0709)<br>N = 506	0.00624<br>(0.0141)<br>N = 506	0.166†<br>(0.0977)<br>N = 490

Note. This table reports estimated treatment effects and robust standard errors in parentheses from ordinary least squares regressions predicting the specified dependent variable using fixed effects for office location, job category, race, and gender. Each regression includes only participants from the demographic subgroup specified.

[a]We only include participants who received an email about talking to a new hire in this intention-to-treat analysis. Some participants in our study were not on the original invite list (e.g., due to people forwarding the training to others), so they did not receive any of the emails for follow-up behavioral measures. For the informal mentoring program and the recognition for excellence program, we can treat these people as nominating zero women; however, because the dependent variable for the audit study is the difference in the willingness to talk to the female versus male new hire, if a participant did not receive any email, they were not randomly assigned to speak to either a female or a male new hire, so we cannot count them in either bucket. We still conduct an intention-to-treat analysis for all participants who received any email about speaking to a new hire.

†, *, **, and *** denote significance at the 10%, 5%, 1%, and 0.1% levels, respectively

TABLE S15

OLS Regressions Predicting Attitudes Pertaining to Women

	**Attitudinal Support for Women** *(Higher Scores Reflect More Supportive Attitudes)*		**Gender Bias Acknowledgment** *(Higher Scores Reflect Smaller Gap)*		**Gender Inclusive Intentions** *(Higher Scores Reflect More Intentions to Be Inclusive)*	
	**Model 1**	**Model 2**	**Model 3**	**Model 4**	**Model 5**	**Model 6**
Treatment	0.246**	0.216*	0.269**	0.299**	0.183†	0.219*
	(0.0901)	(0.0910)	(0.0946)	(0.0969)	(0.0964)	(0.100)
Male Employee	-0.468***	-0.473***	0.294**	0.315**	-0.136	-0.117
	(0.0910)	(0.0928)	(0.0956)	(0.0989)	(0.0979)	(0.103)
U.S. Employee	0.678***	0.362	-0.0465	-0.765*	0.248*	0.288
	(0.109)	(0.290)	(0.114)	(0.308)	(0.116)	(0.316)
Treatment x Male Employee	0.00564	0.0304	-0.0954	-0.128	-0.0258	-0.0225
	(0.112)	(0.112)	(0.117)	(0.119)	(0.119)	(0.123)
Treatment x U.S. Employee	-0.351**	-0.333*	-0.0350	-0.0650	-0.131	-0.161
	(0.133)	(0.133)	(0.140)	(0.141)	(0.142)	(0.146)
Male Employee x U.S. Employee	-0.174	-0.200	-0.0276	-0.0810	-0.0533	-0.0451
	(0.142)	(0.141)	(0.149)	(0.150)	(0.151)	(0.156)
Treatment x Male Employee x U.S. Employee	0.190	0.198	0.0490	0.0904	0.0328	0.0280
	(0.173)	(0.171)	(0.182)	(0.182)	(0.185)	(0.188)
Full Controls Present	No	Yes	No	Yes	No	Yes
**Observations**	2,327	2,327	2,332	2,332	2,282	2,282
**R2**	0.116	0.214	0.0243	0.108	0.0167	0.0723

Note. This table shows ordinary least squares (OLS) regressions predicting attitudinal support for women, gender bias acknowledgment, and gender inclusive intentions after training including all interactions between the treatment, an indicator for the employee being male, and an indicator for the employee being located in the U.S. Standard errors are reported in parentheses. When full controls are present, regressions include fixed effects for office location, job category, and race. Sample sizes vary because we only include employees who filled out each measure.
†, *, **, and *** denote significance at the 10%, 5%, 1%, and 0.1% levels, respectively

TABLE S16

OLS Regressions Predicting Behaviors Pertaining to Women

	**Number of Women Selected for Informal Mentoring**		**Number of Women Nominated for Excellence Award**	
	**Model 1**	**Model 2**	**Model 3**	**Model 4**
Treatment	-0.0919	-0.0886	-0.0114	-0.0135
	(0.0726)	(0.0758)	(0.0141)	(0.0144)
Male Employee	-0.139†	-0.111	-0.0291**	-0.0273*
	(0.0763)	(0.0795)	(0.0101)	(0.0106)
U.S. Employee	-0.208**	-0.428***	-0.0226	-0.0541**
	(0.0759)	(0.106)	(0.0137)	(0.0203)
Treatment x Male Employee	0.0639	0.0599	0.0131	0.0154
	(0.0794)	(0.0840)	(0.0128)	(0.0131)
Treatment x U.S. Employee	0.288**	0.292**	0.0186	0.0203
	(0.0922)	(0.0961)	(0.0185)	(0.0189)
Male Employee x U.S. Employee	0.152†	0.156†	0.0230	0.0244+
	(0.0843)	(0.0889)	(0.0141)	(0.0145)
Treatment x Male Employee x	-0.241**	-0.243*	-0.00338	-0.00677
U.S. Employee	(0.0914)	(0.0980)	(0.0236)	(0.0244)
Full Controls Present	No	Yes	No	Yes
**Observations**	3,011	3,011	3,011	3,011
**R2**	0.0098	0.0655	0.0055	0.0394

Note. This table shows ordinary least squares (OLS) regressions predicting the number of women selected for informal mentoring per consented employee and the number of women recognized for excellence per consented employee after training using all interactions between the treatment, an indicator for the participant being male, and an indicator for the participant being located in the U.S. Robust standard errors are in parentheses and are clustered at the office location level. When full controls are present, regressions include fixed effects for office location, job category, and race.
†, *, **, and *** denote significance at the 10%, 5%, 1%, and 0.1% levels, respectively

TABLE S17

OLS Regressions Predicting Responses to Audit Email

	Willing to Speak to New Hire (Y = 1)	
	**Model 1**	**Model 2**
Treatment	-0.0119<br>(0.0584)	-0.0442<br>(0.0610)
Male Employee	0.0197<br>(0.0575)	-0.0227<br>(0.0592)
U.S. Employee	0.0201<br>(0.0677)	0.0282<br>(0.181)
Female New Hire	-0.0410<br>(0.0677)	-0.0730<br>(0.0697)
Treatment x Male Employee	0.0442<br>(0.0710)	0.0809<br>(0.0735)
Treatment x U.S. Employee	-0.0267<br>(0.0836)	0.0107<br>(0.0860)
Treatment x Female New Hire	0.0851<br>(0.0822)	0.129<br>(0.0848)
Male Employee x U.S. Employee	0.00812<br>(0.0906)	0.0757<br>(0.0919)
Male Employee x Female New Hire	0.0957<br>(0.0830)	0.134<br>(0.0842)
U.S. Employee x Female New Hire	0.0713<br>(0.101)	0.115<br>(0.104)
Treatment x Male Employee x U.S. Employee	-0.0392<br>(0.111)	-0.0886<br>(0.113)
Treatment x Male Employee x Female New Hire	-0.0695<br>(0.102)	-0.113<br>(0.104)
Treatment x U.S. Employee x Female New Hire	0.0536<br>(0.124)	-0.00713<br>(0.127)
Male Employee x U.S. Employee x Female New Hire	-0.0422<br>(0.132)	-0.0952<br>(0.135)
Treatment x Male Employee x U.S. Employee x Female New Hire	-0.100<br>(0.161)	-0.0389<br>(0.165)
Full Controls Present	No	Yes
**Observations**	2,898	2,898
**R2**	0.0109	0.0675

Note. This table shows ordinary least squares (OLS) regressions predicting whether participants respond to an email asking if they would be willing to speak to either a female or male new hire using all interactions between the treatment, an indicator for the participant being male, an indicator for the participant being located in the U.S., and an indicator for the new hire being female. Robust standard errors are in parentheses. When full controls are present, regressions include fixed effects for office location, job category, and race.
†, *, **, and *** denote significance at the 10%, 5%, 1%, and 0.1% levels, respectively

TABLE S18

Summary of Differences Between Treatment and Control Conditions on Attitudes and Behaviors Pertaining to Racial Minorities

	**Difference in Measures (Treatment – Control)**		
**Demographic Subgroup**	**Racial Bias Acknowledgment** *(Higher Scores Reflect Smaller Gap)*	**Number of Racial Minorities Selected for Informal Mentoring**	**Number of Racial Minorities Nominated for Excellence Award**
**Overall**	0.186***<br>(0.0437)<br>N = 2,332	-<br>-<br>-	-<br>-<br>-
**Employees in the U.S.**	0.192**<br>(0.0674)<br>N = 937	0.0511<br>(0.0325)<br>N = 1,159	0.0176†<br>(0.00912)<br>N = 1,159
Whites in the U.S.	0.115<br>(0.0829)<br>N = 579	0.0452<br>(0.0281)<br>N = 688	-0.000134<br>(0.00755)<br>N = 688
Racial Minorities in the U.S.	0.320**<br>(0.115)<br>N = 358	0.0563<br>(0.0685)<br>N = 471	0.0432*<br>(0.0195)<br>N = 471

Note. This table reports differences between the treatment and control conditions, standard errors for these differences in parentheses, and sample sizes for the specified measures split by demographic subgroup. Statistical significance reported is from t-tests.

†, *, **, and *** denote significance at the 10%, 5%, 1%, and 0.1% levels, respectively

TABLE S19

Estimated Treatment Effects on Attitudes and Behaviors Pertaining to Racial Minorities

Using Standard OLS Regression Specifications for Different Subgroups

	**Estimated Treatment Effect**		
**Demographic Subgroup**	**Racial Bias Acknowledgment** *(Higher Scores Reflect Smaller Gap)*	**Number of Racial Minorities Selected for Informal Mentoring**	**Number of Racial Minorities Nominated for Excellence Award**
**Overall**	0.182***<br>(0.0448)<br>N = 2,332	-<br>-<br>-	-<br>-<br>-
**Employees in the U.S.**	0.204**<br>(0.0680)<br>N = 937	0.0469<br>(0.0329)<br>N = 1,159	0.0166†<br>(0.00928)<br>N = 1,159
Whites in the U.S.	0.119<br>(0.0841)<br>N = 579	0.0357<br>(0.0287)<br>N = 688	-0.00135<br>(0.00777)<br>N = 688
Racial Minorities in the U.S.	0.323**<br>(0.121)<br>N = 358	0.0793<br>(0.0721)<br>N = 471	0.0514*<br>(0.0203)<br>N = 471

Note. This table reports estimated treatment effects and standard errors in parentheses from ordinary least squares regressions predicting the specified dependent variable using fixed effects for office location, job category, race, and gender. Each regression includes only participants from the demographic subgroup specified.
†, *, **, and *** denote significance at the 10%, 5%, 1%, and 0.1% levels, respectively

TABLE S20

OLS Regressions Predicting Attitudes and Behaviors Pertaining to Racial Minorities

	**Racial Bias Acknowledgment (Higher Scores Reflect Smaller Gap)**		**Number of Racial Minorities Selected for Informal Mentoring**		**Number of Racial Minorities Nominated for Excellence Award**	
	**Model 1**	**Model 2**	**Model 3**	**Model 4**	**Model 5**	**Model 6**
Treatment	0.320***	0.328***	0.0563	0.0556	0.0432**	0.0449**
	(0.0643)	(0.0642)	(0.0543)	(0.0502)	(0.0126)	(0.0152)
White Employee	0.126	0.678***	-0.114**	0.00496	0.00214	0.0257*
	(0.0974)	(0.0894)	(0.0367)	(0.0432)	(0.00540)	(0.0120)
Treatment x White Employee	-0.205†	-0.195†	-0.0111	-0.0144	-0.0433**	-0.0470**
	(0.115)	(0.104)	(0.0645)	(0.0624)	(0.0127)	(0.0149)
Full Controls Present	No	Yes	No	Yes	No	Yes
**Observations**	937	937	1,159	1,159	1,159	1,159
**R2**	0.011	0.088	0.015	0.056	0.016	0.050

Note. This table shows ordinary least squares (OLS) regressions predicting the difference in racial bias acknowledgment, the number of racial minorities selected for informal mentoring per consented employee, and the number of racial minorities recognized for excellence per consented employee after training using an interaction between the treatment and an indicator for the participant being white. Robust standard errors are in parentheses and are clustered at the office location level for the nomination measures. When full controls are present, regressions include fixed effects for office location, job category, race, and gender. Regressions are limited to U.S. employees.

†, *, **, and *** denote significance at the 10%, 5%, 1%, and 0.1% levels, respectively

TABLE S21

OLS Regressions Examining Whether Women Nominate Junior or Senior Colleagues for Mentoring

	**Number of Women Selected Who Are Junior Colleagues**		**Number of Women Selected Who Are Senior Colleagues**	
	**Model 1**	**Model 2**	**Model 3**	**Model 4**
Treatment	-0.0378	-0.0292	-0.0492	-0.0547
	(0.0450)	(0.0493)	(0.0479)	(0.0487)
Male Employee	-0.0288	-0.0120	-0.110*	-0.0967*
	(0.0480)	(0.0541)	(0.0443)	(0.0425)
U.S. Employee	-0.0873†	-0.265***	-0.115*	-0.162***
	(0.0466)	(0.0758)	(0.0463)	(0.0454)
Treatment x Male Employee	0.0181	0.00238	0.0418	0.0531
	(0.0536)	(0.0589)	(0.0492)	(0.0493)
Treatment x U.S. Employee	0.0899	0.0861	0.187**	0.195**
	(0.0550)	(0.0575)	(0.0619)	(0.0652)
Male Employee x U.S. Employee	0.0407	0.0390	0.111*	0.116*
	(0.0536)	(0.0568)	(0.0489)	(0.0499)
Treatment x Male Employee x U.S. Employee	-0.0695	-0.0569	-0.161*	-0.177**
	(0.0631)	(0.0683)	(0.0626)	(0.0654)
Full Controls Present	No	Yes	No	Yes
**Observations**	3,011	3,011	3,011	3,011
**R2**	0.0028	0.055	0.015	0.068

Note. This table shows ordinary least squares (OLS) regressions predicting the number of women selected for informal mentoring per consented employee who are senior colleagues and who are junior colleagues after training using all interactions between the treatment, an indicator for the participant being male, and an indicator for the participant being located in the U.S. Robust standard errors are in parentheses and are clustered at the office location level. When full controls are present, regressions include fixed effects for office location, job category, and race.

†, *, **, and *** denote significance at the 10%, 5%, 1%, and 0.1% levels, respectively

TABLE S22

OLS Regressions Exploring Whether a Group's Attitudes in the Absence of Training Moderate Diversity Training's Effects on Attitudes and Behavior Using Country-Gender Subgroups

	**Attitudinal Support for Women** *(Higher Scores Reflect More Supportive Attitudes)*		**Gender Bias Acknowledgment** *(Higher Scores Reflect Smaller Gap)*		**Gender Inclusive Intentions** *(Higher Scores Reflect More Inclusion)*		**Number of Women Selected for Informal Mentoring**		**Number of Women Nominated for Excellence Award**		**Willing to Speak to New Hire (Y = 1)**	
	**Model 1**	**Model 2**	**Model 3**	**Model 4**	**Model 5**	**Model 6**	**Model 7**	**Model 8**	**Model 9**	**Model 10**	**Model 11**	**Model 12**
Treatment	0.122**	0.124**	0.220***	0.218***	0.122**	0.144**	0.0160	0.0181	0.00315	0.00270	-0.00574	-0.0107
	(0.0404)	(0.0410)	(0.0437)	(0.0441)	(0.0445)	(0.0455)	(0.0253)	(0.0257)	(0.00518)	(0.00549)	(0.0264)	(0.0268)
Proxy for Pre-Training Attitudes	0.628***	0.420***	-0.227***	-0.0260	0.188***	0.0936	-0.0679†	-0.0804	0.00407	-0.00505	0.00848	0.0753*
	(0.0367)	(0.0682)	(0.0397)	(0.0733)	(0.0412)	(0.0764)	(0.0384)	(0.0681)	(0.00316)	(0.00754)	(0.0225)	(0.0350)
Treatment x Proxy for Pre-Training	-0.286***	-0.273***	0.171***	0.138**	-0.129*	-0.121*	0.111**	0.112*	-0.00264	-0.00206	-0.0371	-0.0321
Attitudes	(0.0459)	(0.0470)	(0.0497)	(0.0505)	(0.0512)	(0.0530)	(0.0407)	(0.0440)	(0.00455)	(0.00481)	(0.0289)	(0.0293)
Treatment x Proxy for Pre-Training	-	-	-	-	-	-	-	-	-	-	0.0359	0.0365
Attitudes x Female New Hire	-	-	-	-	-	-	-	-	-	-	(0.0433)	(0.0444)
Full Controls Present	No	Yes	No	Yes	No	Yes	No	Yes	No	Yes	No	Yes
**Observations**	2,276	2,276	2,281	2,281	2,232	2,232	2,935	2,935	2,935	2,935	2,834	2,834
**R2**	0.169	0.227	0.025	0.108	0.0148	0.0763	0.005	0.0656	0.0006	0.0365	0.0057	0.0618

Note. This table shows ordinary least squares (OLS) regressions predicting attitudes and behaviors after training using the treatment, a proxy for pre-training levels of attitudinal support for women that has been standardized, and the interaction between these two variables. For the audit measure (Models 11 and 12), the new hire's gender was randomized, so all of these terms are interacted with an indicator for the new hire being female and robust standard errors are used. For the nomination measures, standard errors are clustered by office location. Standard errors are in parentheses. When controls are present, regressions include fixed effects for office location, tenure, race, and gender.

†, *, **, and *** denote significance at the 10%, 5%, 1%, and 0.1% levels, respectively

TABLE S23

OLS Regressions Exploring Whether a Group's Attitudes in the Absence of Training Moderate Diversity Training's Effects on Attitudes and Behavior Using Office Location-Gender Subgroups

	**Attitudinal Support for Women** *(Higher Scores Reflect More Supportive Attitudes)*		**Gender Bias Acknowledgment** *(Higher Scores Reflect Smaller Gap)*		**Gender Inclusive Intentions** *(Higher Scores Reflect More Inclusion)*		**Number of Women Selected for Informal Mentoring**		**Number of Women Nominated for Excellence Award**		**Willing to Speak to New Hire (Y = 1)**	
	**Model 1**	**Model 2**	**Model 3**	**Model 4**	**Model 5**	**Model 6**	**Model 7**	**Model 8**	**Model 9**	**Model 10**	**Model 11**	**Model 12**
Treatment	0.0802*	0.0850*	0.238***	0.233***	0.121**	0.137**	0.0228	0.0228	0.00410	0.004111	-0.00957	-0.0125
	(0.0406)	(0.0410)	(0.0445)	(0.0446)	(0.0452)	(0.0459)	(0.0263)	(0.0260)	(0.00518)	(0.00548)	(0.0271)	(0.0273)
Proxy for Pre-Training Attitudes	1.000***	0.791***	-0.301***	-0.0665	0.273***	0.207*	-0.0759	-0.0480	0.00234	-0.00930	0.0104	0.0916†
	(0.0518)	(0.0896)	(0.0568)	(0.0973)	(0.0589)	(0.102)	(0.0553)	(0.0777)	(0.00479)	(0.00994)	(0.0342)	(0.0482)
Treatment x Proxy for Pre-Training	-0.555***	-0.510***	0.198**	0.156*	-0.178*	-0.159*	0.123*	0.125*	-0.000798	-0.00129	-0.0518	-0.0490
Attitudes	(0.0650)	(0.0661)	(0.0712)	(0.0718)	(0.0732)	(0.0752)	(0.0561)	(0.0593)	(0.00643)	(0.00671)	(0.0428)	(0.0429)
Treatment x Proxy for Pre-Training	-	-	-	-	-	-	-	-	-	-	0.0657	0.0858
Attitudes x Female New Hire	-	-	-	-	-	-	-	-	-	-	(0.0624)	(0.0634)
Full Controls Present	No	Yes	No	Yes	No	Yes	No	Yes	No	Yes	No	Yes
**Observations**	2,210	2,210	2,215	2,215	2,167	2,167	2,847	2,847	2,847	2,847	2,752	2,752
**R2**	0.190	0.243	0.0252	0.108	0.0165	0.0735	0.0031	0.0631	0.0004	0.0295	0.0065	0.0619

Note. This table shows ordinary least squares (OLS) regressions predicting attitudes and behaviors after training using the treatment, a proxy for pre-training levels of attitudinal support for women that has been standardized, and the interaction between these two variables. For the audit measure (Models 11 and 12), the new hire's gender was randomized, so all of these terms are interacted with an indicator for the new hire being female and robust standard errors are used. For the nomination measures, standard errors are clustered by office location. Standard errors are in parentheses. When controls are present, regressions include fixed effects for office location, tenure, race, and gender.

†, *, **, and *** denote significance at the 10%, 5%, 1%, and 0.1% levels, respectively

TABLE S24

OLS Regressions Exploring Whether a Group's Attitudes in the Absence of Training Moderate Diversity Training's Effects on Attitudes and Behavior Using Office Location-Gender Subgroups Limited to Employees in the United States

	**Attitudinal Support for Women** *(Higher Scores Reflect More Supportive Attitudes)*		**Gender Bias Acknowledgment** *(Higher Scores Reflect Smaller Gap)*		**Gender Inclusive Intentions** *(Higher Scores Reflect More Inclusion)*		**Number of Women Selected for Informal Mentoring**		**Number of Women Nominated for Excellence Award**		**Willing to Speak to New Hire (Y = 1)**	
	**Model 1**	**Model 2**	**Model 3**	**Model 4**	**Model 5**	**Model 6**	**Model 7**	**Model 8**	**Model 9**	**Model 10**	**Model 11**	**Model 12**
Treatment	0.154*	0.167*	0.180*	0.164*	0.0502	0.0543	0.0775*	0.0736*	0.0174*	0.0171†	0.0116	-0.00585
	(0.0757)	(0.0774)	(0.0806)	(0.0816)	(0.0852)	(0.0878)	(0.0308)	(0.0344)	(0.00826)	(0.00879)	(0.0529)	(0.0533)
Proxy for Pre-Training Attitudes	0.987***	0.835***	-0.227†	0.0771	0.175	0.0727	0.0444	0.0200	0.00486	-0.0139	0.0819	0.103
	(0.116)	(0.194)	(0.123)	(0.205)	(0.131)	(0.230)	(0.0746)	(0.121)	(0.00727)	(0.0177)	(0.0818)	(0.110)
Treatment x Proxy for Pre-Training	-0.581***	-0.573***	0.127	0.127	0.0196	0.0133	0.0777	0.103	-0.0159	-0.0162	-0.142	-0.104
Attitudes	(0.144)	(0.147)	(0.153)	(0.154)	(0.162)	(0.167)	(0.0834)	(0.0735)	(0.0149)	(0.0148)	(0.101)	(0.101)
Treatment x Proxy for Pre-Training	-	-	-	-	-	-	-	-	-	-	0.300*	0.287*
Attitudes x Female New Hire	-	-	-	-	-	-	-	-	-	-	(0.141)	(0.143)
Full Controls Present	No	Yes	No	Yes	No	Yes	No	Yes	No	Yes	No	Yes
**Observations**	919	919	923	923	908	908	1,141	1,141	1,141	1,141	1,115	1,115
**R2**	0.095	0.139	0.0165	0.0822	0.0071	0.0408	0.0097	0.0571	0.0029	0.0309	0.0163	0.0534

Note. This table shows ordinary least squares (OLS) regressions predicting attitudes and behaviors after training using the treatment, a proxy for pre-training levels of attitudinal support for women that has been standardized, and the interaction between these two variables. For the audit measure (Models 11 and 12), the new hire's gender was randomized, so all of these terms are interacted with an indicator for the new hire being female and robust standard errors are used. For the nomination measures, standard errors are clustered by office location. Standard errors are in parentheses. When controls are present, regressions include fixed effects for office location, tenure, race, and gender.

†, *, **, and *** denote significance at the 10%, 5%, 1%, and 0.1% levels, respectively

TABLE S25

Correlation Matrix for Variables Collected During Situational Judgment Test Validation

	1	2	3	4	5	6	7	8	9	10
**1. Situational Judgment Test**	1.00									
**2. Modern Sexism**	-.189***	1.00								
**3. Ambivalent Sexism**	-.235***	.614***	1.00							
**4. Benevolent Sexism**	-.187***	.160**	.716***	1.00						
**5. Hostile Sexism**	-.177**	.732***	.808***	.197***	1.00					
**6. Social Dominance Orientation**	-.241***	.642***	.527***	.216**	.571***	1.00				
**7. Authoritarian Aggression**	-.213***	.442***	.665***	.545***	.484***	.441***	1.00			
**8. Conservatism**	.143*	-.559***	-.586***	-.375***	-.494***	-.541***	-.618***	1.00		
**9. Gender Bias Acknowledgment**	.217***	-.5489***	-.324***	-0.13*	-.364***	-.424***	-.272***	.320***	1.00	
**10. Racial Bias Acknowledgment**	0.178**	-.297***	-.136*	.035	-.195***	-.315***	-.118*	.160**	.620***	1.00

*, **, and *** denote significance at the 5%, 1%, and 0.1% levels, respectively

TABLE S26

Hierarchical Linear Models Predicting Financial Contribution to a Women's Cause for Situational Judgment Test Validation: Attitude Measures

	**Model 1**	**Model 2**	**Model 3**
Gender Bias Acknowledgment	-0.001	0.000	-0.001
	(0.004)	(0.004)	(0.004)
Modern Sexism	-0.009	-0.008	-0.007
	(0.006)	(0.006)	(0.006)
Hostile Sexism	0.002	0.001	0.002
	(0.006)	(0.006)	(0.006)
Benevolent Sexism	0.010	0.009	0.012
	(0.006)	(0.006)	(0.007)
Gender-Career IAT Score		-0.024	-0.021
		(0.021)	(0.021)
SJT			0.003*
			(0.002)
**Observations**	243	243	243
**R^2**	0.024	0.029	0.046

Note. This table shows hierarchical linear regressions predicting the amount of an offered financial bonus that participants contributed to a women's professional development organization. Standard errors are reported in parentheses.

*, **, and *** denote significance at the 5%, 1%, and 0.1% levels, respectively

TABLE S27

Hierarchical Linear Models Predicting Financial Contribution to a Women's Cause for Situational Judgment Test Validation: Personality Measures

	**Model 1**	**Model 2**	**Model 3**
Actively Open-Minded Thinking	-0.009	-0.016*	-0.020**
	(0.006)	(0.007)	(0.007)
Agreeableness		0.014**	0.012*
		(0.005)	(0.005)
Extraversion		-0.006	-0.005
		(0.004)	(0.004)
Conscientiousness		-0.001	0.000
		(0.005)	(0.005)
Intellect		0.003	0.003
		(0.006)	(0.006)
Neuroticism		-0.002	-0.001
		(0.004)	(0.004)
SJT			0.004*
			(0.002)
**Observations**	243	243	243
**R^2**	0.007	0.048	0.067

Note. This table shows hierarchical linear regressions predicting the amount of an offered financial bonus that participants contributed to a women's professional development organization. Standard errors are reported in parentheses.

*, **, and *** denote significance at the 5%, 1%, and 0.1% levels, respectively

TABLE S28

Hierarchical Linear Models Predicting Financial Contribution to a Women's Cause for Situational Judgment Test Validation: Attitude and Personality Measures

	**Model 1**	**Model 2**	**Model 3**	**Model 4**
Gender Bias Acknowledgment	-0.001	0.000	-0.002	-0.002
	(0.004)	(0.004)	(0.004)	(0.004)
Modern Sexism	-0.009	-0.008	-0.010	-0.009
	(0.006)	(0.006)	(0.006)	(0.006)
Hostile Sexism	0.002	0.001	0.004	0.003
	(0.006)	(0.006)	(0.006)	(0.006)
Benevolent Sexism	0.010	0.009	0.004	0.006
	(0.006)	(0.006)	(0.007)	(0.007)
Gender-Career IAT Score		-0.024	-0.016	-0.015
		(0.021)	(0.021)	(0.021)
Actively Open-Minded Thinking			-0.016	-0.018*
			(0.008)	(0.008)
Agreeableness			0.012*	0.011*
			(0.005)	(0.005)
Extraversion			-0.006	-0.005
			(0.004)	(0.004)
Conscientiousness			0.000	0.001
			(0.005)	(0.005)
Intellect			0.002	0.002
			(0.006)	(0.006)
Neuroticism			-0.003	-0.002
			(0.004)	(0.004)
SJT				0.004
				(0.002)*
**Observations**	243	243	243	243
**R^2**	0.024	0.029	0.065	0.083

Note. This table shows hierarchical linear regressions predicting the amount of an offered financial bonus that participants contributed to a women's professional development organization. Standard errors are reported in parentheses.

*, **, and *** denote significance at the 5%, 1%, and 0.1% levels, respectively

SCREENSHOTS OF INTERVENTION

Gender-Bias Condition:

Table of Contents Screen Displayed at the Outset of the Gender-Bias Condition:

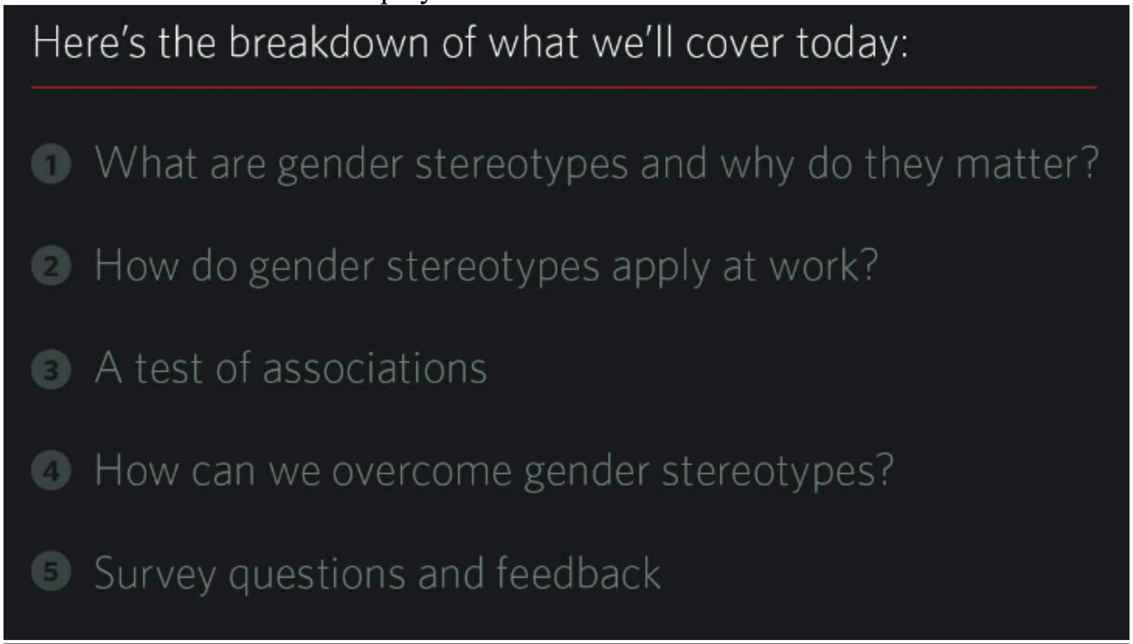

Example of Content from Section 1 about Gender Stereotypes:

How does categorization affect the way we think about men and women?

Gender stereotypes are thoughts or beliefs about the qualities that men and women possess. These beliefs allow us to quickly, though not always accurately, process information about others.

An example of a gender stereotype is a belief that women are caring.

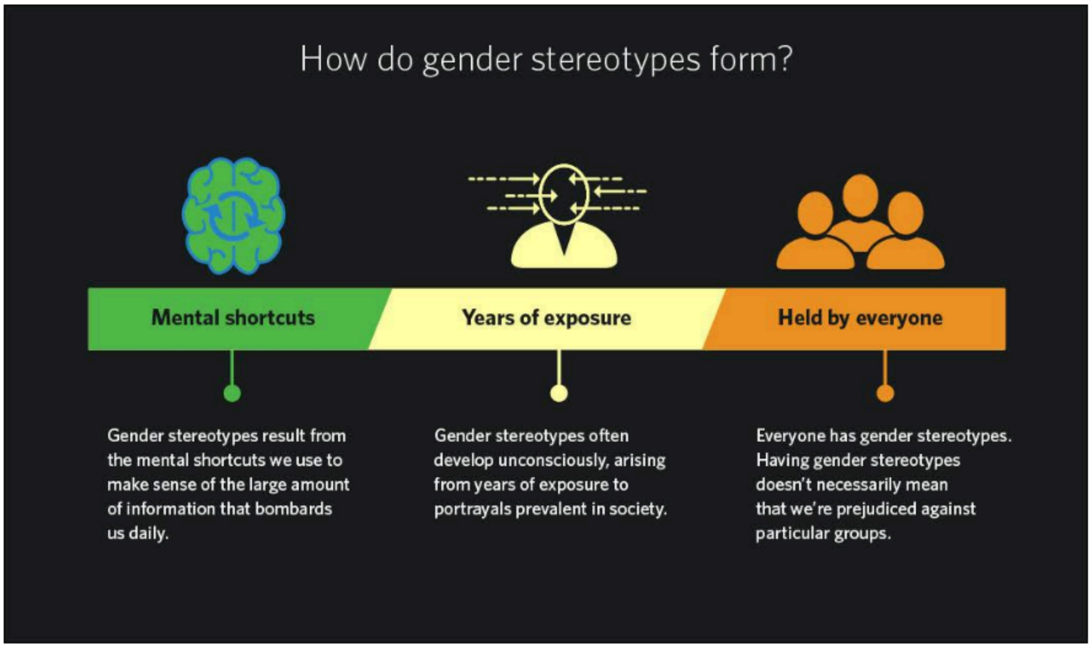

Example of Content from Section 2 about How Gender Stereotypes Apply at Work:

How do gender stereotypes affect professional life?

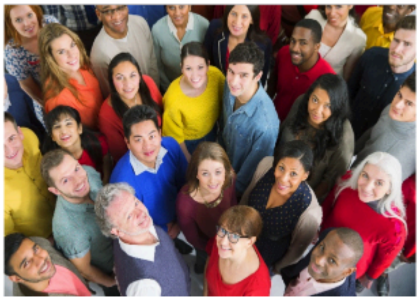

Gender stereotypes can influence the way we act toward others, even when we don't realize it. This "unconscious bias" can lead to undesirable work outcomes.

A field experiment found that White female undergraduates were significantly less likely than White males to receive a response from faculty members in science and math departments requesting an opportunity to meet in the future to discuss their interest in pursuing a PhD.

Understanding that unconscious bias exists and affects career outcomes is the first step in overcoming it to create more inclusive work environments. Next, we'll review scientific studies demonstrating unconscious gender bias in the workplace.

Source: Milkman, K. L., Akinola, M., & Chugh, D. (2015). *Journal of Applied Psychology*, 100, 1678-1712.

Example of Content from Section 3 about A Test of Associations:

The Implicit Association Test

Next, you'll be asked to take an Implicit Association Test (IAT). Your score on the test is based on how long it takes to categorize concepts with stereotypes. For example, if someone is faster at categorizing words when categorizing Men with Science and Women with Humanities than the reverse (Men with Humanities and Women with Science), we would say that they implicitly and automatically associate Men with Science.

If the IAT shows an automatic association, it does not mean that a test taker is prejudiced! Such associations are often held subconsciously as a result of repeated exposure to stereotypes. People's implicit associations may well conflict with their explicit beliefs.

Example of Content from Section 4 about How We Can Overcome Stereotypes:

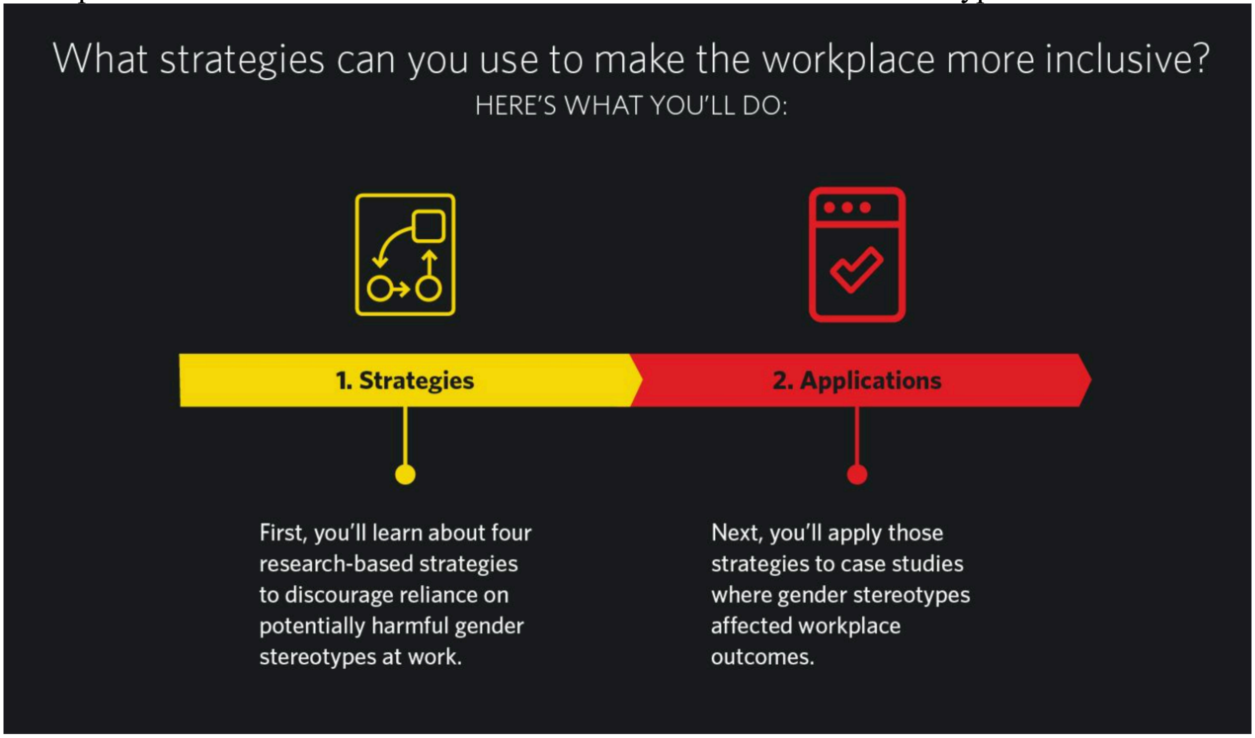

Example of Content from Section 5 about Survey Questions:

You will now be asked to answer questions about the extent to which you exhibit a number of established psychological tendencies that all people show to some extent.

Gender Stereotyping: Many studies have found that people often make automatic assumptions about people based on their gender. For example, people associate men with technology and women with housework.

Please answer the following questions:

To what extent do you believe that you exhibit gender stereotyping?

Not at all<br>1	2	3	Somewhat<br>4	5	6	Very Much<br>7
○	○	○	○	○	○	○

To what extent do you believe that other people exhibit gender stereotyping?

Not at all<br>1	2	3	Somewhat<br>4	5	6	Very Much<br>7
○	○	○	○	○	○	○

General-Bias Condition:

Table of Contents Screen Displayed at the Outset of the General-Bias Condition:

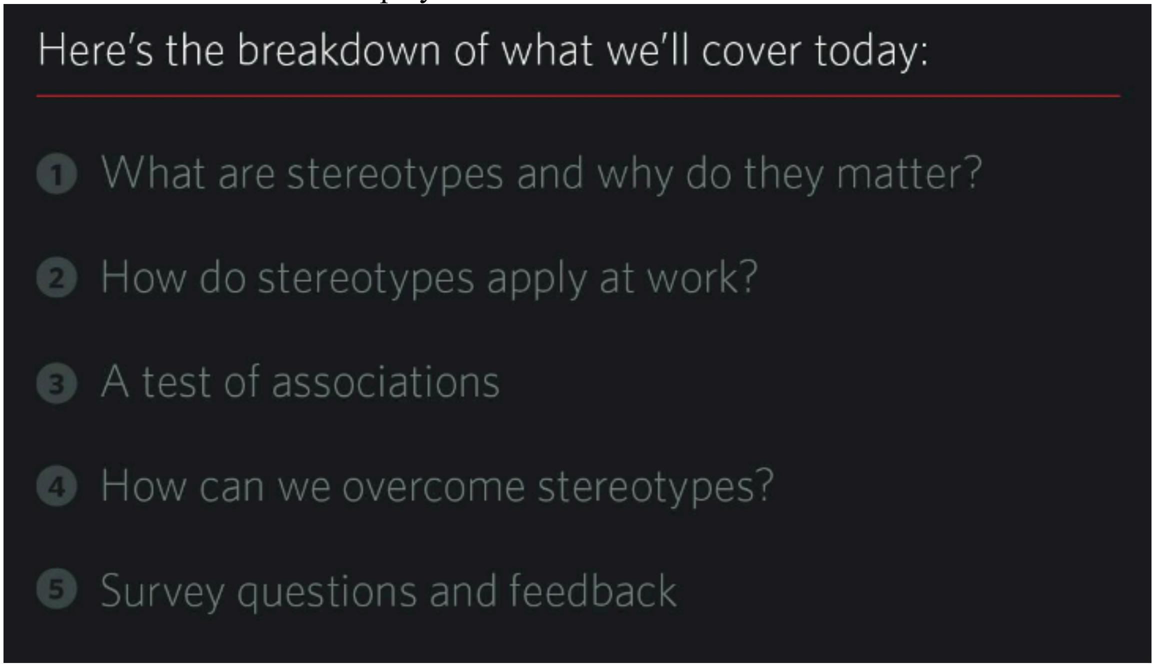

Example of Content from Section 1 about Stereotypes:

How does categorization affect the way we think about other people?

Stereotypes are thoughts or beliefs about the qualities that members of different groups possess. These beliefs allow us to quickly, though not always accurately, process information about others.

An example of a stereotype is a belief that young people are good with technology.

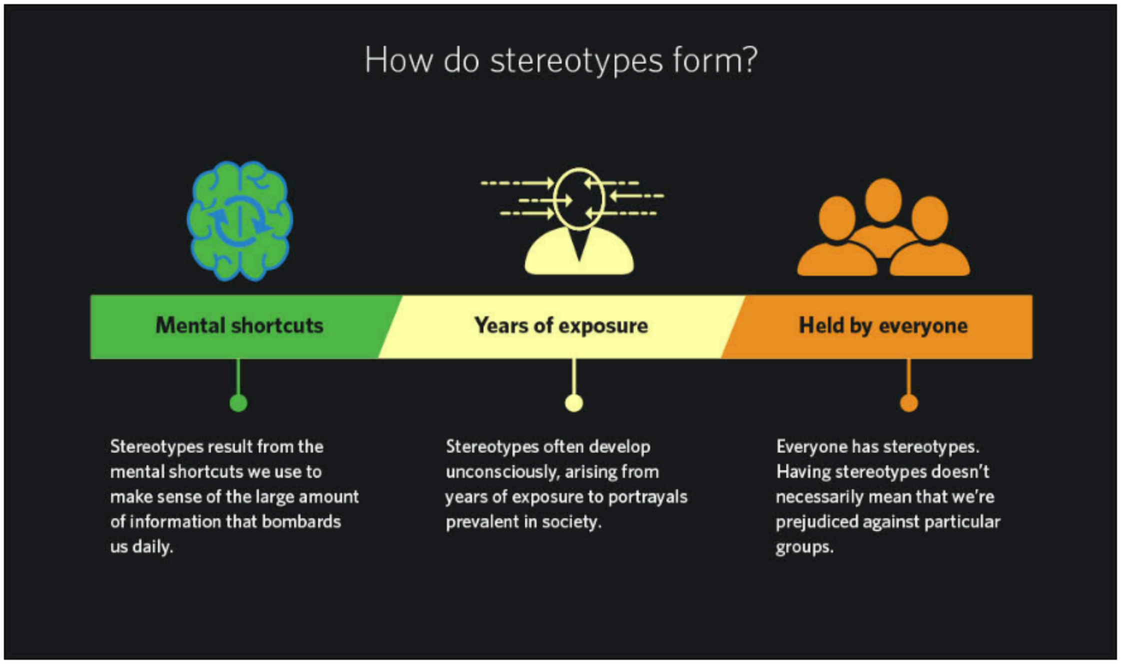

Example of Content from Section 2 about How Stereotypes Apply at Work:

How do stereotypes affect professional life?

Stereotypes can influence the way we act toward others, even when we don't realize it. This "unconscious bias" can lead to undesirable work outcomes.

A field experiment found that Indian undergraduates were significantly less likely than White males to receive a response from faculty members in science and math departments requesting an opportunity to meet in the future to discuss their interest in pursuing a PhD.

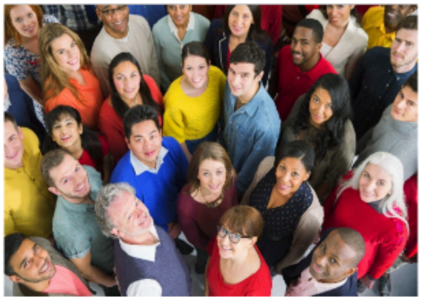

Understanding that unconscious bias exists and affects career outcomes is the first step in overcoming it to create more inclusive work environments. Next, we'll review scientific studies demonstrating unconscious bias in the workplace.

Source: Milkman, K. L., Akinola, M., & Chugh, D. (2015). Journal of Applied Psychology, 100, 1678-1712.

Example of Content from Section 3 about A Test of Associations:

The Implicit Association Test

Next, you'll be asked to take an Implicit Association Test (IAT). Your score on the test is based on how long it takes to categorize concepts with stereotypes. For example, if someone is faster at categorizing words when categorizing Straight Men with Masculine and Gay Men with Feminine than the reverse (Straight Men with Feminine and Gay Men with Masculine), we would say that they implicitly and automatically associate Straight Men with Masculine.

If the IAT shows an automatic association, it does not mean that a test taker is prejudiced! Such associations are often held subconsciously as a result of repeated exposure to stereotypes. People's implicit associations may well conflict with their explicit beliefs.

Example of Content from Section 4 about How We Can Overcome Stereotypes:

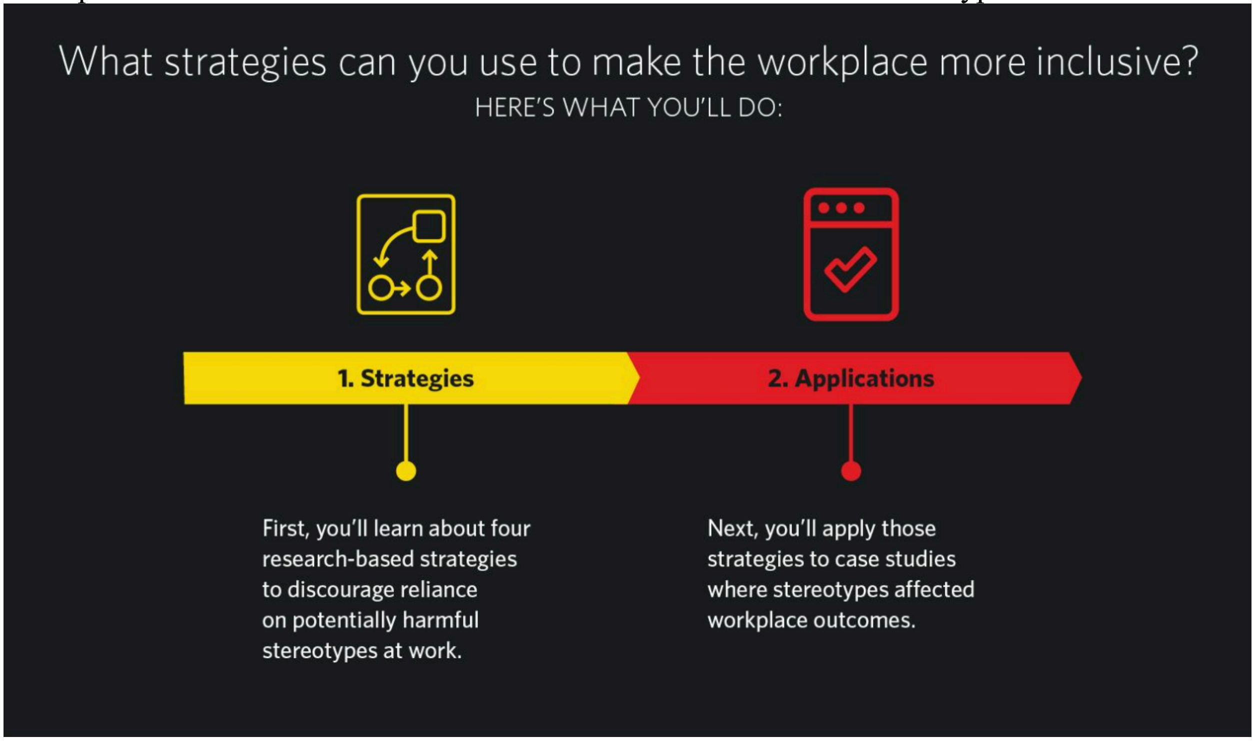

Example of Content from Section 5 about Survey Questions:

You will now be asked to answer questions about the extent to which you exhibit a number of established psychological tendencies that all people show to some extent.

Gender Stereotyping: Many studies have found that people often make automatic assumptions about people based on their gender. For example, people associate men with technology and women with housework.

Please answer the following questions:

To what extent do you believe that you exhibit gender stereotyping?

Not at all 1	2	3	Somewhat 4	5	6	Very Much 7
○	○	○	○	○	○	○

To what extent do you believe that other people exhibit gender stereotyping?

Not at all 1	2	3	Somewhat 4	5	6	Very Much 7
○	○	○	○	○	○	○

Control Condition:
Table of Contents Screen Displayed at the Outset of the Control Condition:

Here's what we'll cover today:

1. Why is inclusive leadership important?
2. What makes teams more inclusive?
3. What strategies can you use to build psychological safety?
4. A test of listening skills
5. Survey questions and feedback

Example of Content from Section 1 about Why Inclusive Leadership is Important:

How can leaders make their teams more intelligent?

The central finding of Google's quest to build the perfect team was that *how* teams work together is more important than *who* is on the team in the first place.

Both the employee experience and ultimately business outcomes are influenced by the extent to which team members feel safe to take risks and like they can depend on their colleagues, among other factors.

As a result, one of the important roles a leader can play is to create an inclusive environment for everyone on the team.

Example of Content from Section 2 about How to Make Teams More Inclusive:

How can leaders make their teams more intelligent?

Collective intelligence is the ability of a single team to perform well on a wide variety of different tasks.

Studies led by Professor Anita Woolley show that this "c factor" is not merely the product of the average or maximum intelligence of individual team members, but instead appears to be driven more by how teams work together. In particular, "groups where a few people dominated the conversation were less collectively intelligent than those with a more equal distribution of conversational turn-taking."

Part of becoming an inclusive leader is learning how to create group norms and a team environment where everyone can contribute.

SOURCE: Woolley, A. W., Chabris, C. F., Pentland, A., Hashmi, N., & Malone, T. W. (2010). Evidence for a collective intelligence factor in the performance of human groups. *Science*, *330*(6004), 686-688.

Example of Content from Section 3 about Strategies for Building Psychological Safety:

How can you promote psychological safety in your teams?

The three different strategies for increasing psychological safety on your teams form an easy-to-remember acronym:

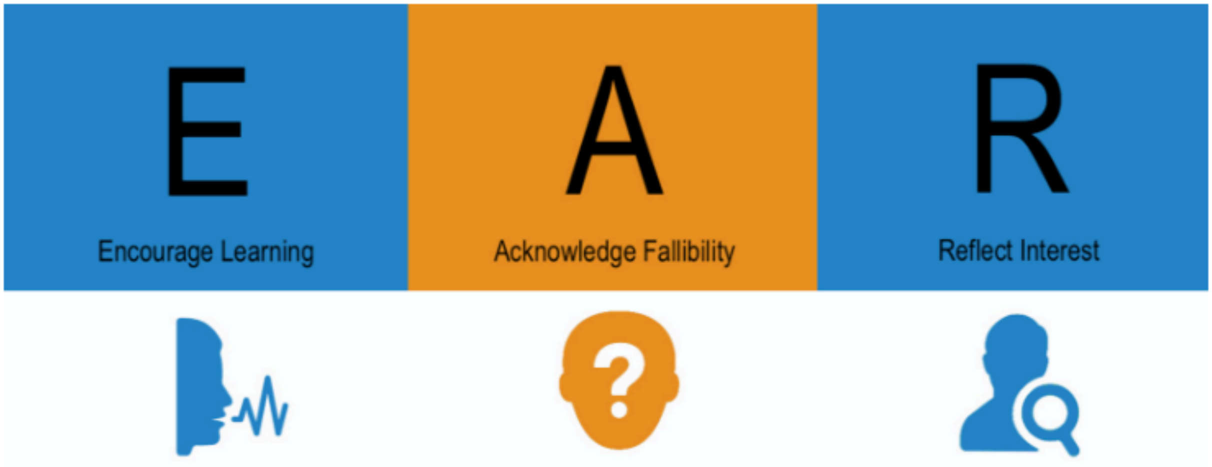

Example of Interactive Content from Section 4:

Test your listening skills

A recurring theme throughout the strategies for building psychological safety is that to be an inclusive leader, you need to hone your ability to listen to your team. Listening makes it easier to see opportunities to encourage learning, to acknowledge fallibility, and to reflect interest in your team.

You will now have the opportunity to take a version of the Active Empathic Listening (AEL) Scale that was used in the previously mentioned study of salespeople. After answering the questions on the next page, you will receive your overall AEL score and more information about the three dimensions of Active Empathic Listening.

Example of Content from Section 5 about Survey Questions:

You will now be asked to answer questions about the extent to which you exhibit a number of established psychological tendencies that all people show to some extent.

Gender Stereotyping: Many studies have found that people often make automatic assumptions about people based on their gender. For example, people associate men with technology and women with housework.

Please answer the following questions:

To what extent do you believe that you exhibit gender stereotyping?

Not at all<br>1	2	3	Somewhat<br>4	5	6	Very Much<br>7
○	○	○	○	○	○	○

To what extent do you believe that other people exhibit gender stereotyping?

Not at all<br>1	2	3	Somewhat<br>4	5	6	Very Much<br>7
○	○	○	○	○	○	○

REFERENCES

Baron, J., Scott, S., Fincher, K., & Metz, S. E. (2015). Why does the Cognitive Reflection Test (sometimes) predict utilitarian moral judgment (and other things)? *Journal of Applied Research in Memory and Cognition*, *4*(3), 265–284.

Bowles, H. R., Babcock, L., & Lai, L. (2007). Social incentives for gender differences in the propensity to initiate negotiations: Sometimes it does hurt to ask. *Organizational Behavior and Human Decision Processes*, *103*(1), 84–103.

Brandt, M. J. (2011). Sexism and gender inequality across 57 societies. *Psychological Science*, *22*(11), 1413–1418.

Buhrmester, M., Kwang, T., & Gosling, S. D. (2011). Amazon's Mechanical Turk a new source of inexpensive, yet high-quality, data? *Perspectives on Psychological Science*, *6*(1), 3–5.

Davis, N. J., & Robinson, R. V. (1991). Men's and women's consciousness of gender inequality: Austria, West Germany, Great Britain, and the United States. *American Sociological Review*, 72–84.

Donnellan, M. B., Oswald, F. L., Baird, B. M., & Lucas, R. E. (2006). The mini-IPIP scales: tiny-yet-effective measures of the Big Five factors of personality. *Psychological Assessment*, *18*(2), 192.

Duguid, M. M., & Thomas-Hunt, M. C. (2015). Condoning stereotyping? How awareness of stereotyping prevalence impacts expression of stereotypes. *Journal of Applied Psychology*, *100*(2), 343.

Glick, P., & Fiske, S. T. (1996). The ambivalent sexism inventory: Differentiating hostile and benevolent sexism. *Journal of Personality and Social Psychology*, *70*(3), 491.

Greenwald, A. G., McGhee, D. E., & Schwartz, J. L. (1998). Measuring individual differences in implicit cognition: the implicit association test. *Journal of Personality and Social Psychology*, *74*(6), 1464.

Hooper, A. C., Cullen, M. J., & Sackett, P. R. (2006). Operational threats to the use of SJTs: Faking, coaching, and retesting issues. *Situational Judgment Tests: Theory, Measurement, and Application*, 205–232.

Inglehart, R., & Norris, P. (2003). *Rising tide: Gender equality and cultural change around the world*. Cambridge University Press.

Lievens, F., Peeters, H., & Schollaert, E. (2008). Situational judgment tests: A review of recent research. *Personnel Review*, *37*(4), 426–441.

Oostrom, J. K., De Soete, B., & Lievens, F. (2015). Situational judgment testing. *Employee Recruitment, Selection, and Assessment: Contemporary Issues for Theory and Practice*, 172–189.

Orne, M. T. (1962). On the social psychology of the psychological experiment: With particular reference to demand characteristics and their implications. *American Psychologist*, *17*(11), 776.

Pietri, E. S., Moss-Racusin, C. A., Dovidio, J. F., Guha, D., Roussos, G., Brescoll, V. L., & Handelsman, J. (2017). Using video to increase gender bias literacy toward women in science. *Psychology of Women Quarterly*, *41*(2), 175–196.

Pratto, F., Sidanius, J., Stallworth, L. M., & Malle, B. F. (1994). Social dominance orientation: A personality variable predicting social and political attitudes. *Journal of Personality and Social Psychology*, *67*(4), 741.

Rattazzi, A. M. M., Bobbio, A., & Canova, L. (2007). A short version of the Right-Wing Authoritarianism (RWA) Scale. *Personality and Individual Differences*, *43*(5), 1223–1234.

Rogelberg, S. G., & Stanton, J. M. (2007). *Introduction: Understanding and dealing with organizational survey nonresponse*. Sage Publications Sage CA: Los Angeles, CA.

Rudman, L. A., & Glick, P. (1999). Feminized management and backlash toward agentic women: the hidden costs to women of a kinder, gentler image of middle managers. *Journal of Personality and Social Psychology*, *77*(5), 1004.

Stemig, M. S., Sackett, P. R., & Lievens, F. (2015). Effects of organizationally endorsed coaching on performance and validity of situational judgment tests. *International Journal of Selection and Assessment*, *23*(2), 174–181.

Swim, J. K., Aikin, K. J., Hall, W. S., & Hunter, B. A. (1995). Sexism and racism: Old-fashioned and modern prejudices. *Journal of Personality and Social Psychology*, *68*(2), 199.

Tversky, A., & Kahneman, D. (1974). Judgment under uncertainty: Heuristics and biases. *Science*, *185*(4157), 1124–1131.

Weekley, J. A., & Ployhart, R. E. (2013). *Situational judgment tests: Theory, measurement, and application*. Psychology Press.

Zizzo, D. J. (2010). Experimenter demand effects in economic experiments. *Experimental Economics*, *13*(1), 75–98.
